# Topographic Organization of Correlation Along the Longitudinal and Transverse Axes in Rat Hippocampal CA3 Due to Excitatory Afferents

**DOI:** 10.3389/fncom.2020.588881

**Published:** 2020-11-20

**Authors:** Gene J. Yu, Jean-Marie C. Bouteiller, Theodore W. Berger

**Affiliations:** Department of Biomedical Engineering, Center for Neural Engineering, University of Southern California, Los Angeles, CA, United States

**Keywords:** correlation, CA3, large-scale model, connectivity, neuronal network, hippocampus, topography

## Abstract

The topographic organization of afferents to the hippocampal CA3 subfield are well-studied, but their role in influencing the spatiotemporal dynamics of population activity is not understood. Using a large-scale, computational neuronal network model of the entorhinal-dentate-CA3 system, the effects of the perforant path, mossy fibers, and associational system on the propagation and transformation of network spiking patterns were investigated. A correlation map was constructed to characterize the spatial structure and temporal evolution of pairwise correlations which underlie the emergent patterns found in the population activity. The topographic organization of the associational system gave rise to changes in the spatial correlation structure along the longitudinal and transverse axes of the CA3. The resulting gradients may provide a basis for the known functional organization observed in hippocampus.

## Introduction

The architecture and connectivity of rat hippocampus has been intensely studied, revealing a prominent topographic organization within the highly complex and tortuous structure of the hippocampal anatomy. Despite a thorough characterization of the macroscale, mesoscale, and microscale connectivity of rat hippocampus, the contributions of the architecture of the afferent and efferent projections to the organization of population dynamics has yet to be fully understood. This is partly due to the difficulty in interpreting and integrating the results of the key studies, many of which were performed several decades ago, into a single comprehensive model. The technical difficulty in recreating these studies with either older or more modern methods limit further characterizations of the microscale and mesoscale topography. Few computational models of neural systems have attempted to represent their full geometrical, or at least up to an extent at which the mesoscale contributions to population activity can be observed (Schneider et al., 2012; Markram et al., 2015; Hendrickson et al., 2016; Billeh et al., 2020). Yet, these types of anatomical-scale models are necessary to explore the contributions of topographically organized connectivity on the spatio-temporal dynamics of their respective neural systems.

At a basic level, connectivity determines the spatial arrangement of postsynaptic activation given a presynaptic spike resulting in a correlation across neurons, i.e., a spatially organized correlation. Pairwise spike correlations between neurons have been shown to capture much of the statistical properties of a single neuron and provide a measure for studying the properties of population activity (Helias et al., 2014; Dettner et al., 2016). Weak pairwise correlations have been demonstrated to give rise to emergent spatiotemporal structures in population activity (Halliday, 2000; Schneidman et al., 2006; Kriener et al., 2009; Renart et al., 2010; Senk et al., 2018; Yu et al., 2018).

The relation between connectivity and spatially organized correlation is due to the spatial distribution of an axon and the sparsity/density of its connectivity which then determine the amount of input overlap/input sharing that occurs among neurons. Theoretical studies have characterized the role of input sharing in determining the correlation that a postsynaptic population exhibits and the propagation of the correlation through multiple layers (Kumar et al., 2010; Rosenbaum et al., 2011, 2016; Darshan et al., 2018). Such studies have also revealed how the interactions between feedforward and recurrent inhibitory circuits nonlinearly affect the spatial structure of correlation. Beyond spatial correlation, the temporal correlation can also be considered in the correlation structure which is determined by the electrophysiology of the postsynaptic neuron and the synaptic properties (Tetzlaff et al., 2008; Hong et al., 2012; Chan et al., 2016; Yu et al., 2018). Given these principles, we investigated the population dynamics and spatiotemporal correlation structure that resulted from different types of connectivity in a hippocampus-specific context.

Using an entorhinal-dentate network, we had previously revealed a role of anatomically-constrained connectivity in organizing random inputs into spatially and temporally dense regions of activity called clusters (Hendrickson et al., 2016; Yu et al., 2018). The spatial properties of the clusters were found to be influenced by the anatomy of the axonal projections, i.e., the spatial extent of the axon terminal field. This previous work was limited to exploring the effect of a single feedforward projection on a single neural population. In the present work, the entorhinal-dentate work was expanded to investigate (1) how spatio-temporal patterns within the dentate gyrus would be preserved when propagated to the CA3 subfield, (2) how multiple feedforward projections would interact to influence the spatio-temporal pattern of the CA3, and (3) how a recurrent excitatory projection, i.e., the associational system, would further transform the activity.

**Figure 1:**
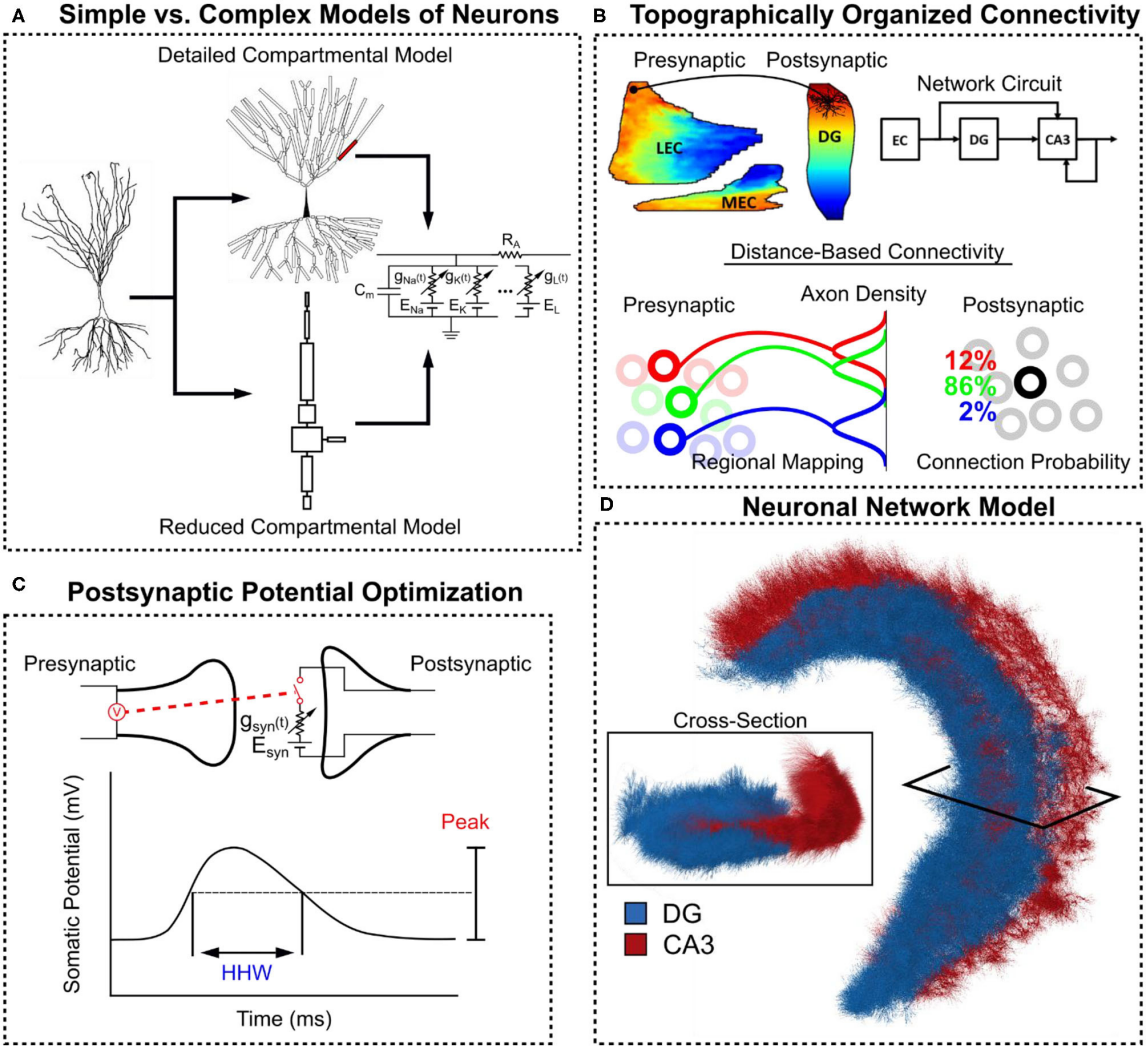
Overview of methods in constructing the large-scale hippocampal model. **(A)** Compartmental models of CA3 pyramidal cells with Hodgkin-Huxley style dynamics were constructed. A detailed version with realistic morphology was converted into a reduced equivalent compartment model. **(B)** Anatomical data was used to define a topographically-organized, spatially-dependent connectivity. **(C)** The postsynaptic potential for each pre-post synapse type was characterized using their peak value and their half-height width (HHW). **(D)** A three-dimensional hippocampal model consisting of the dentate gyrus and CA3 was constructed for this study.

A large-scale entorhinal-dentate-CA3 neuronal network model with spatially-dependent and topographically-organized connectivity was constructed that encompasses the full geometric extent of a rat hippocampus using compartmental models of neurons (Figure 1). From the entorhinal-dentate network, the perforant path projection and dentate mossy fibers were included to connect the entorhinal cortex and dentate gyrus to the CA3, and the recurrent associational system was added. By computing a spatio-temporal correlation map, a heterogeneous correlation structure was discovered which varied in amplitude and shape along both the longitudinal and transverse spatial axes of the CA3 and further evolve in time.

## Materials and Methods

### Neuron Models

**Figure 2:**
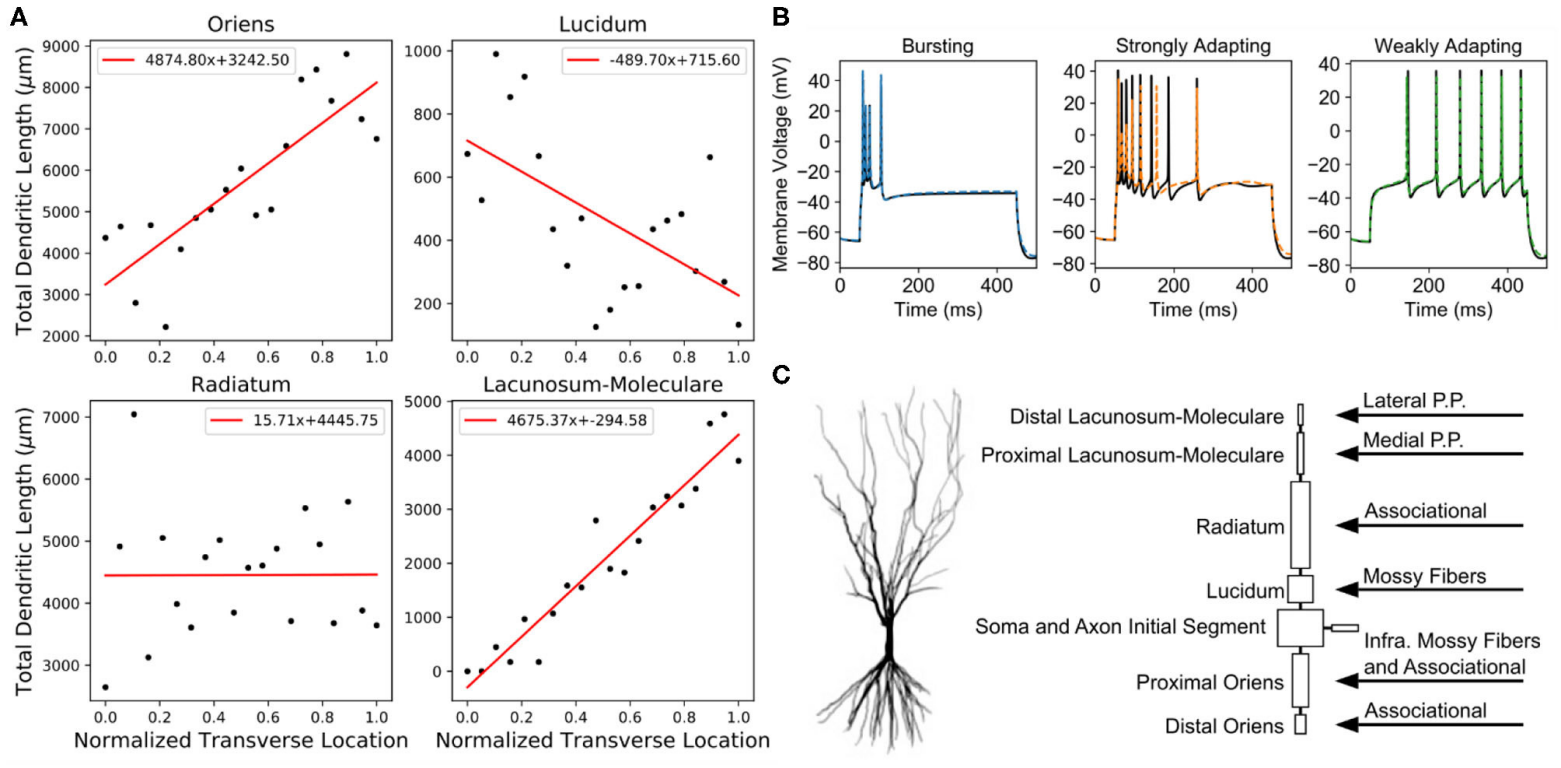
Anatomical details and spiking behavior of CA3 pyramidal cell models. **(A)** The total dendritic length varied based on the transverse position of the model within the CA3 subfield. These lengths and the synaptic densities in each layer (Table 1) were used to determine the numbers of each input type that a model received. **(B)** After the reduced models (dashed colored lines) preserved the different spiking behaviors as the original models (solid black lines). **(C)** The original morphology was reduced to eight compartments. On the right, the specific regions that received a particular input type are denoted.

The CA3 pyramidal cell is the principal neuron of the CA3, and the basis of the CA3 pyramidal cell models used in the present work originated from a study in which three major firing types were discovered: bursting, strongly adapting, and weakly adapting (Hemond et al., 2008). They published three models with biophysical parameters and spiking behavior that best represented *in vivo* recordings of CA3 pyramidal cells that demonstrated the different firing types. For the three models, the biophysical parameters had been distributed upon a single, morphological reconstruction of the apical and basal dendrites of a CA3 pyramidal cell. The models contained the following ion channels: sodium, delayed-rectifier K^+^, A-type K^+^, D-type K^+^, M-type K^+^, T-type Ca^2+^, N-type Ca^2+^, L-type Ca^2+^, calcium-dependent K^+^ (CaGK), calcium-dependent K^+^ (BK), HCN, and leak channels (see Supplementary Tables 1–3). In the present work, the morphology of the models from Hemond et al. (2008) was simplified using an algorithm that used circuit theory to combine compartments connected in series and in parallel to create simplified equivalent circuit representations of complex morphologies (Marasco et al., 2012). The algorithm was used in the current work to construct simplified models while preserving the firing behavior exhibited by the original models (Figure 2C). The simplified models contained eight compartments corresponding to a compartment for each layer upon which input is received and reduced simulation times by a factor of 20. Model parameters are summarized in the Supplementary Tables 1–4. The computational models were simulated using NEURON v7.5 and scripted using Python 2.7.

The entorhinal-dentate network used in the present study was the same as described in Yu et al. (2018, 2019) and is extensively described there. Dentate granule cells were represented using a simplified morphology that was constructed using the same technique as for the CA3 pyramidal cell models (Marasco et al., 2012). Entorhinal cortical cells were represented using a renewal process that consisted of a homogeneous Poisson process with an exponentially-decaying refractory period with a time constant of 35 ms.

**Table 1:** Number of inputs received by the CA3 pyramidal cells and EPSP properties.

	**Lacunosum distal (LEC)**	**Lacunosum proximal (MEC)**	**Radiatum (associational)**	**Oriens (associational)**
Synaptic Density^a^	0.63	0.63	3.61	3.15
Number of Inputs (Proximal)	0	0	11,241	7,147
Max. Number of Inputs (Distal)	1,658	1,105	11,281	17,893

*The proximal vs. distal inputs are based on the multiplication of the synaptic density and the total dendritic length within the relevant layer. Between the proximal and distal ends, the numbers of inputs change linearly*.

a *Megias et al. (2001)*.

The entorhinal-dentate-CA3 network was comprised of 112,000 entorhinal cortical cells, 120,000 dentate granule cells, and 25,000 CA3 pyramidal cells which represents one-tenth of the full number of dentate granule cells and CA3 pyramidal cells within the rat hippocampus (Mulders et al., 1997). Each simulation represented 5 s of real-time at a time step of 0.025 ms and was run using 100 cores from Dual Intel Xeon 2.4 GHz CPUs resulting in a wall-time of approximately 4 h per simulation. Each core was allocated 2 GB of RAM for a total of 200 GB of RAM per simulation. The simulations were performed using the computing resources provided by the Center for Advanced Research Computing at the University of Southern California.

### Topography of Afferent Inputs to CA3 Pyramidal Cells

#### Hippocampal Anatomy

**Figure 3:**
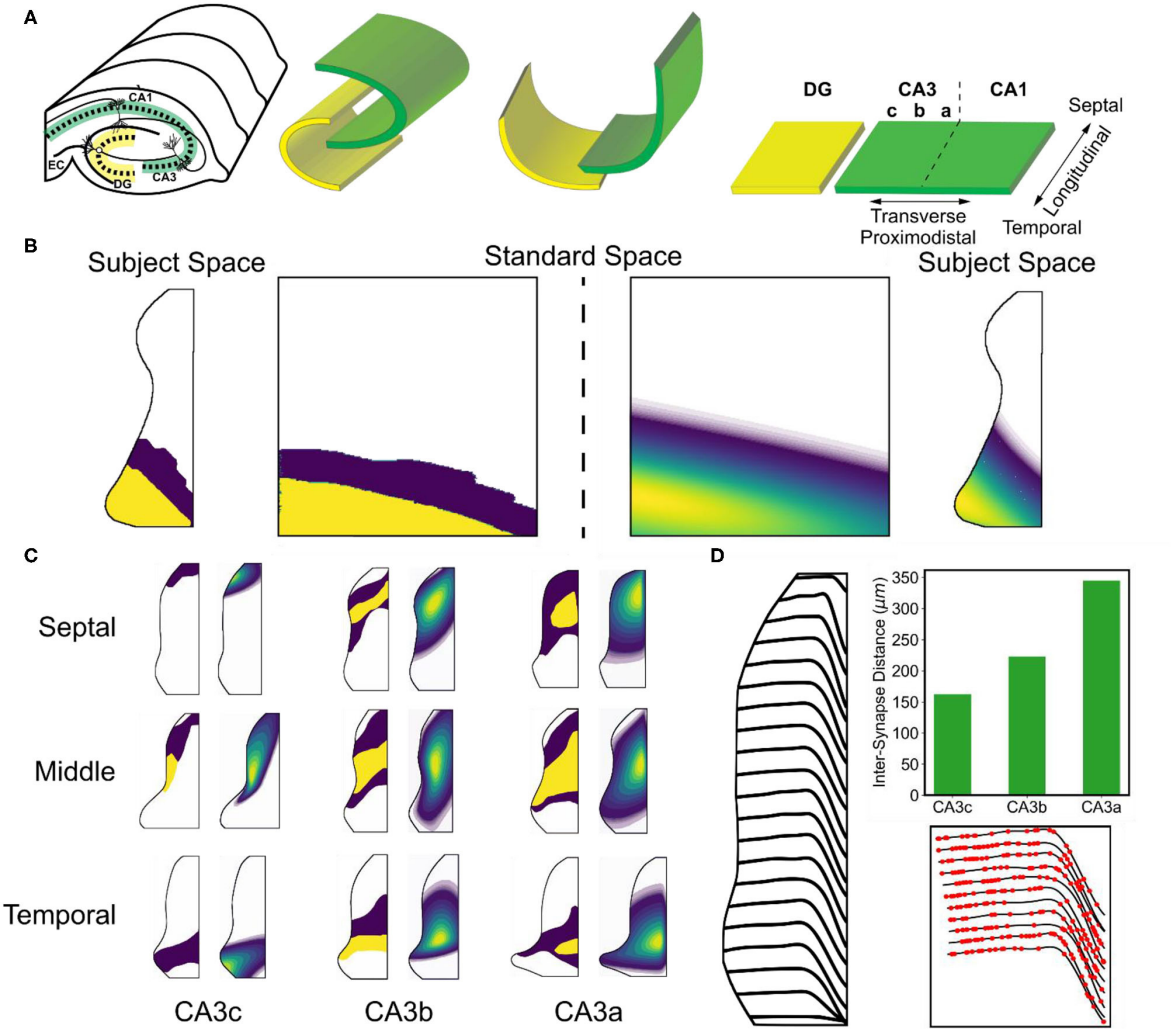
Overview of mossy fiber and associational topography. **(A)** A conceptual diagram depicts how a flattened representation of the hippocampus can be made from the original 3D structure. The transverse axis refers to the proximodistal axis. The longitudinal axis refers to the septotemporal/ dorsoventral axes. **(B)** The original data that revealed the organization of the associational system was reported using two intensity values. The data was mapped onto a standard space, fit to a parameterized equation, and then remapped back into subject space. **(C)** Examples of the original data and resulting fits are shown. **(D)** The trajectories of the mossy fibers are shown in the CA3 subfield. The top right subplot indicates the change inter-synapse distance that occurs along the mossy fiber. The bottom right subplot shows example synapse locations.

To describe the CA3 network model, some background regarding the structure of the hippocampus must be given, and common terminology to describe the hippocampal anatomy must be established. Briefly, the rat hippocampus is organized into three areas: the dentate gyrus, CA3 subfield, and CA1 subfield (Figure 3A). The simplified trisynaptic circuit of the hippocampus is a predominantly feedforward pathway that begins in the entorhinal cortex and describes the propagation of activity from entorhinal cortex, to dentate gyrus, to CA3, and finally to CA1 (Andersen, 1975). There are many more details to the full description of the circuits within the hippocampus such as back projections and the CA2, but the simplified trisynaptic circuit captures the major organization of the hippocampus.

The hippocampus is a three-dimensional structure, and the curved nature of the layers do not allow positions to easily be described using three-dimensional cartesian coordinate systems. Therefore, neuroanatomists developed a technique to unfold and flatten the structure to describe the anatomy using a two-dimensional coordinate system (Figure 3A). The longitudinal axis can generally refer to the dorso-ventral axis, septo-temporal axis, or y-axis of the hippocampus. The transverse axis can generally refer to the proximodistal axis or x-axis of the hippocampus. The proximodistal axis within CA3 refers to position with respect to the dentate gyrus, and the CA3 has been commonly divided into three subregions along this axis. The CA3c, CA3b, and CA3a are organized with the CA3c being most proximal to dentate gyrus and CA3a being most distal to dentate gyrus.

#### Anatomically-Constrained Mesoscale and Microscale Connectivity

The major intrahippocampal afferents to CA3 pyramidal cells were considered in this study: the lateral perforant path input, the medial perforant path input, mossy fiber input, and the associational input. Anatomical data was used to define and constrain the topography of the various projections. The topography describes the regional mapping between layers/subfields of the hippocampal formation. Relevant data include anterograde and retrograde tracer injection studies which can reveal the relation between position within a hippocampal subfield and the region within the postsynaptic area to which axons are sent (anterograde) or the region within the presynaptic area from which axons are received (retrograde). Another crucial aspect of the anatomy is the spatial distribution of the axon terminal field within the postsynaptic area. Under the assumption that a greater axonal density results in a larger number of connections, the axonal distribution was converted into a probability distribution with a higher density corresponding to a higher probability of connection. Thus, connectivity was stochastically generated by first defining the regional mapping between the position of a presynaptic neuron and the postsynaptic region to which axons are sent. Then, the axonal density was used to define a spatial constraint resulting in a spatially dependent connectivity. The topographic regional mapping corresponds to mesoscale connectivity, and the resulting connections based on the axonal density correspond to the microscale connectivity.

Axons were not explicitly represented in the models but were represented as a propagation delay based on the path length between the presynaptic and postsynaptic neurons and the conduction velocity. Conduction velocities of 0.32 m/s, 0.27 m/s, and 0.39 m/s were used for the perforant path (Tielen et al., 1981), mossy fibers (Kress et al., 2008), and associational system (Andersen et al., 2000), respectively. The anatomical data and methods for quantifying them that are described below were initially introduced in earlier work (Yu et al., 2014, 2015).

The perforant path refers to the projection arising from the entorhinal cortex that are sent to the dentate gyrus and CA3 and is divided into the lateral and medial perforant path based on their origin from the lateral and medial areas of the entorhinal cortex. They initially synapse onto the dentate gyrus before continuing onwards and making a monosynaptic connection with the CA3 (Yeckel and Berger, 1990). Within the dentate gyrus and CA3, the lateral and medial perforant paths terminate on different strata which, for the CA3, are the distal and proximal lacunosum-moleculare, respectively. Because the same axons that synapse in the dentate gyrus continue to the CA3, the topographical map from entorhinal cortex to dentate gyrus was used to describe the mapping from entorhinal cortex to CA3. The data used to describe the entorhinal-dentate topography came from the series of retrograde tracer studies (Dolorfo and Amaral, 1998), and the extent of the axon terminal field along the longitudinal axis was reported to be 1–1.5 mm (Tamamaki, 1997). A Gaussian distribution was used to represent the connection probability of a perforant path axon terminal field. The resulting map predicts a longitudinal organization of the entorhinal projection to dentate gyrus (Figure 1B). A detailed description of the data and method for extracting the topography are described previously in Hendrickson et al. (2016) and Yu et al. (2019). To summarize this method, a workflow was developed to digitize the data, map the results of the injection onto a standard space, perform averaging when relevant within the standard space, and finally remap the averaged data onto a chosen subject rat space.

The mossy fibers describe the axons that are sent by dentate granule cells to the CA3. Each mossy fiber can be generally characterized as a single fiber which initially stays within the same longitudinal level from which it originates and then travels through the CA3 predominantly along the transverse axis for the first two-thirds (i.e., within CA3c and CA3b) before turning toward the temporal pole of the longitudinal axis within CA3a (Figure 3D) (Acsády et al., 1998). The fiber trajectories were estimated using data published in Swanson et al. (1978) by measuring the deviation of the fiber with respect to the longitudinal level of origin as it traversed the proximodistal extent of the CA3. The deviations as a function of longitudinal origin were interpolated using a cubic b-splines fit to represent a smooth change in fiber trajectory. To generate the fibers, noise was added to the points representing each fiber trajectory create variable fibers.

The longitudinal and transverse organization of the associational system was most thoroughly revealed by Ishizuka et al. (1990) using anterograde tracers. The tracer was injected into one of nine areas within the CA3 which roughly covered a 3 × 3 grid with injections within the CA3c, CA3b, and CA3a as well as the septal, middle, and temporal levels. The resulting distribution of tracer represents the density and spatial extent to which axons were sent. Density in the study was represented using three qualitative levels: a zero level, a low density level, and a high density level. Similar to the entorhinal-dentate/CA3 topography, the data were digitized and mapped onto a standard space (Figure 3B). Within the standard space, distribution of labeling was parameterized using a two-dimensional skew gaussian equation. The parameters of the equation could then be interpolated/extrapolated to estimate the distribution of CA3 associational axons for areas that were not covered by an injection (Figure 3C).

### Numbers of Synapses

The final step to generating connectivity is to define the numbers of connections that are possible. There are two method by which the number of connections could be constrained. From a postsynaptic perspective, the number of inputs that could be received for a presynaptic population can be estimated by using spine count information which could be obtained by using spine density and dendritic length measurements. Due to highly stratified nature of the CA3 afferents, the number of inputs for each afferent could be estimated by calculating the total number of spines for the different layers to which the afferents project. The second method for constraining the number of inputs is to use the presynaptic population's axon measurements. The bouton density and the axon length can be used to estimate the number of connections that a presynaptic neuron forms with a postsynaptic population. The strata to which the various projections are restricted are summarized in Figure 2.

The total dendritic length of each strata within CA3 change along the proximodistal axis as revealed by Ishizuka et al. (1995). In general, the total dendritic length is smallest proximally, and it is largest distally (Figure 2A). The spine density within each stratum are not well studied for CA3 pyramidal cells. Rather, spine densities have been meticulously characterized for CA1 pyramidal cells and were used to estimate the numbers of synapses for CA3 pyramidal cells (Megias et al., 2001). The total synapse numbers are summarized in Table 1. Using these calculations, the number of inputs for the perforant path and associational projections were constrained for the model.

Because the synaptic density within the stratum lucidum was not characterized in CA1, the second method of using the presynaptic axon properties was used to constrain the number of inputs for the mossy fibers. The inter-synapse distance had been measured for mossy fibers which revealed that the inter-synapse distance changed as the fiber moved from CA3c to CA3b to CA3a (Acsády et al., 1998). Using a Poisson process, the locations of mossy fiber synapses along the fiber were estimated using the reported mean values of 162 ± 12.6 μm in CA3c, 223 ± 19.3 μm in CA3b, and 345 ± 27.5 μm in CA3a (Figure 3D). Mossy fibers originating in the suprapyramidal blade of the dentate gyrus were restricted to the stratum lucidum. Mossy fibers originating in the infrapyramidal blade were restricted to the proximal stratum oriens within CA3c before moving into the stratum lucidum for the CA3b and CA3a.

Having defined the topography, spatially dependent connection probabilities, and the numbers of connections the connectivity of the network could be stochastically generated. For the postsynaptic method, the connection probabilities for each presynaptic neuron for a given afferent were collected for each CA3 pyramidal cell. The connection probabilities were normalized, and a presynaptic neuron was randomly selected until the total number of connections for that particular afferent was achieved. For the presynaptic method, a postsynaptic neuron was randomly selected for each synapse location. A postsynaptic neuron within 30 μm of the synapse was considered based on measurements performed by Acsády et al. (1998). The distribution of the number of mossy fiber inputs is found in Supplementary Figure 1.

### Synapse Models

Neuron communication in the model was mediated exclusively through synapse-like processes. The synapse was modeled as a deterministic process in which an action potential activates a change in synaptic conductance. The time-course of the synaptic conductance was represented using a double exponential function for AMPA receptors (Kleppe and Robinson, 1999) and a triple exponential function for NMDA receptors. The NMDA receptor was additionally modulated by a sigmoidal function to capture the magnesium-related voltage dependence of the receptor (Jahr and Stevens, 1990; Zador et al., 1990).

$$\begin{array}{l} g_{AMPAR}\left(t\right)\propto e^{\frac{-t}{\tau_{2}}}-e^{\frac{-t}{\tau_{1}}},\;\tau_{2}>\tau_{1}\\ g_{NMDAR}\left(t,v\right)\propto \frac{w\cdot e^{\frac{-t}{\tau_{3}}}+\left(1-w\right)\cdot e^{\frac{-t}{\tau_{2}}}-e^{\frac{-t}{\tau_{1}}}}{1+e^{-0.062v\cdot \left[{Mg}^{2+}\right]/3.57}},\;\tau_{3}>\tau_{2}>\tau_{1} \end{array}$$

The *t* and *v* variables correspond to time in milliseconds and membrane potential in millivolts. The τ variables are time constants that control the time-course of the waveform. The *w* variable for the NMDA receptor is a weighting variable constrained within (0, 1). The equations are normalized using the peak value of the exponential functions, i.e., ignoring the denominator for the NMDA receptor. This can be solved analytically for the AMPA receptor by setting the derivative to zero. For the NMDA receptor, the solution was empirically derived after setting the derivative to zero and finding the intersection of the left- and right-hand sides of the equation. The normalized synaptic conductance equations are then multiplied by a factor corresponding to the synaptic weight. The synaptic weights are constrained such that the resulting excitatory postsynaptic potential (EPSP) recorded from the soma match those reported from unitary synaptic release experiments for the relevant presynaptic-postsynaptic synapse pairings. The time constants for the AMPA receptor were similarly separately constrained such that the half-height width of resulting somatic EPSP matched the experimentally reported values (Table 2). The parameters for the synapses are summarized in Supplementary Tables 5–8.

**Table 2:** EPSP properties.

	**Lacunosum distal (LEC)**	**Lacunosum proximal (MEC)**	**Radiatum (associational)**	**Lucidum (mossy fiber)**	**Oriens proximal (mossy fiber)**	**Oriens distal (associational)**
Peak (mV)	0.30^a^	0.30^a^	0.30^a^	3.2^b^	3.2^b^	0.30^a^
HHW (ms)	46.1^a^	46.1^a^	40.9^a^	135^c^	135^c^	38.0^a^

a *Perez-Rosello et al. (2011)*.

b *Lawrence et al. (2003)*.

c *Scanziani et al. (1993)*.

### Three-Dimensional Space-Time Correlation Maps

**Figure 4:**
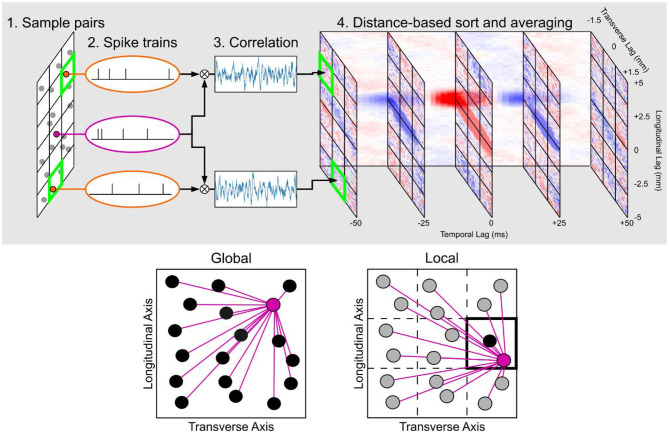
Summary of the construction of the space-time correlation maps. (**Top row**) The workflow in calculating pairwise spike correlations and placing them in a matrix that is organized by the longitudinal, transverse, and temporal lags. (**Bottom row**) Global vs. local space-time correlation map. In the global map, all neuron pairs were considered to construct an average correlation map to represent all neurons that were included to compute the map. In the local map, only neuron pairs with at least one neuron within the chosen section were used in the calculation which results in a correlation map that is specific to the section to which the neuron pairs were constrained. In the local map example highlighted in the figure, the resulting correlation map would be representative for the neurons within the section located in the second row and third column.

The spatial and temporal correlation structure of the network was constructed by computing the pairwise correlation of the spiking activity for all neuron pairs and averaging the normalized cross-correlations of neuron pairs that were located at the same relative distance (Figure 4). The normalized cross-correlation was computed by binning the spike times of the neurons and using the following equation:

$$\begin{array}{l} \left(x⋆y\right)\left[n\right]=\frac{1}{\sigma_{x}\sigma_{y}}\overset{N-1}{\underset{m=0}{\sum }}\frac{1}{L_{m}}\left(x\left[m\right]-\mu_{x}\right)\left(y\left[m+n\right]-\mu_{y}\right) \end{array}$$

for which the ⋆ operator represents correlation, *x* and *y* correspond to the binary spike trains, σ is the standard deviation of the spike trains, μ is the mean of the spike trains, *N* is the total length of the spike train, and *L*_*m*_ is the size of the overlap between the signals while they are being shifted.

A three-dimensional matrix was constructed with an axis corresponding to time, an axis for the longitudinal distance between the neuron pair, and an axis for the transverse distance between the neuron pair. The temporal resolution for binning the spikes was 1 ms. The spatial resolution for the longitudinal and transverse axes was 0.1 mm.

There were two types of space-time correlation maps that were generated for this study (Figure 4). The first type was the global map which computed the space-time correlation map for all possible neuron pairs. The global map then represents the average correlation structure for all principal neurons within a hippocampal subfield. The second type of map was the local map which divided the CA3 into a 3 × 3 grid of longitudinal and transverse sections based on the CA3a, CA3b, and CA3c subdivisions along the transverse axis and the septal, middle, and temporal subdivisions along the longitudinal axis. Local maps were specific to each of these sections and were computed using neuron pairs only if at least one of the neurons was located in the corresponding longitudinal/transverse section. This constraint caused the resulting space-time correlation map to be representative of a smaller population of neurons, as defined by the longitudinal/transverse position. In contrast to the global map which considered every neuron pair, the local map provided a more granular characterization of the correlation map a local map to represent each of the nine longitudinal/transverse sections.

## Results

Input to the entorhinal-dentate-CA3 network was primarily provided by the entorhinal cortex, and the spiking activity of each entorhinal neuron was represented with a renewal process comprised of a Poisson process with an exponentially decaying refractory period that modified the spiking probability after the generation of a spike. The mean firing rate of the Poisson process was 5 Hz. The resulting input had a uniform power density in the frequency domain and was spatially and temporally uncorrelated. Though the random input does not contain behavioral or spatial information, the mean firing rate was determined based on the mean firing rate of the grid cells modeled in Yu et al. (2019) and served as a control to eliminate any correlation that may arise due to a common physiological/behavioral drive.

The simulations described throughout the results can be organized based on the afferent projections that were present and the activity of the dentate granule cells. For the different afferent projections, there was the perforant path (PP-CA3) model which includes only the entorhinal projection, the mossy fiber (MF-CA3) model which includes only the mossy fiber projection, the perforant path-mossy fiber (PP-MF-CA3) model which includes both perforant path and mossy fiber projections, and the perforant path-mossy-fiber-assocational (PP-MF-A-CA3) model which includes the perforant path, mossy fiber, and associational projections. As described in the previous paragraph, the entorhinal cortex only generated random input. However, the dentate granule cell activity was generated using two methods. The first method uses the entorhinal activity and the dentate gyrus network model to generate the dentate granule cell activity and represents the natural dentate response to entorhinal activity. This method introduces a weak spatial and temporal correlation to the dentate granule cell activity due to the topographic connectivity. The second method represents the dentate granule cell activity using a renewal process with a mean firing rate of 0.62 Hz which was the mean firing rate of the dentate granule cells due to entorhinal input. Therefore, the key difference between the first and second methods for dentate granule cell activity was the presence of an inherent spatial and temporal correlation in the activity using the first method and the absence of a correlation in the activity using the second method. These differences in terms of the model are denoted using as weakly correlated mossy fiber (wcMF) or random mossy fiber (rMF).

Simulations were initially performed with synaptic weights that were constrained to elicit the appropriate EPSP peak values, and additional simulations were performed that multiplied the original synaptic weights with scalar factors. The perforant path and mossy fibers each had their synaptic weights modified by factors of 0.5, 1, 2, 3, and 5 with 1 corresponding to the original synaptic weight.

### Spiking Activity and Global Correlation Maps

The longitudinal axis of the hippocampus is much larger than the transverse axes of the hippocampal regions. Additionally, there is a significant longitudinal organization to the topography between regions. These details have supported the presentation of hippocampal activity along the longitudinal axis. The following subsections present the raster plots of spiking activity and the corresponding space-time correlation maps as functions of longitudinal position and time with the same scale to demonstrate the similarities between the spatio-temporal patterns of activity and the space-time correlation map. The slice of the space-time correlation map was taken at a transverse lag of 0 mm, and the color maps were thresholded to 30% of the maximum value to better visualize the weaker negative correlations.

#### Entorhinal Perforant Path Projection (PP-CA3 and PP-DG Models)

**Figure 5:**
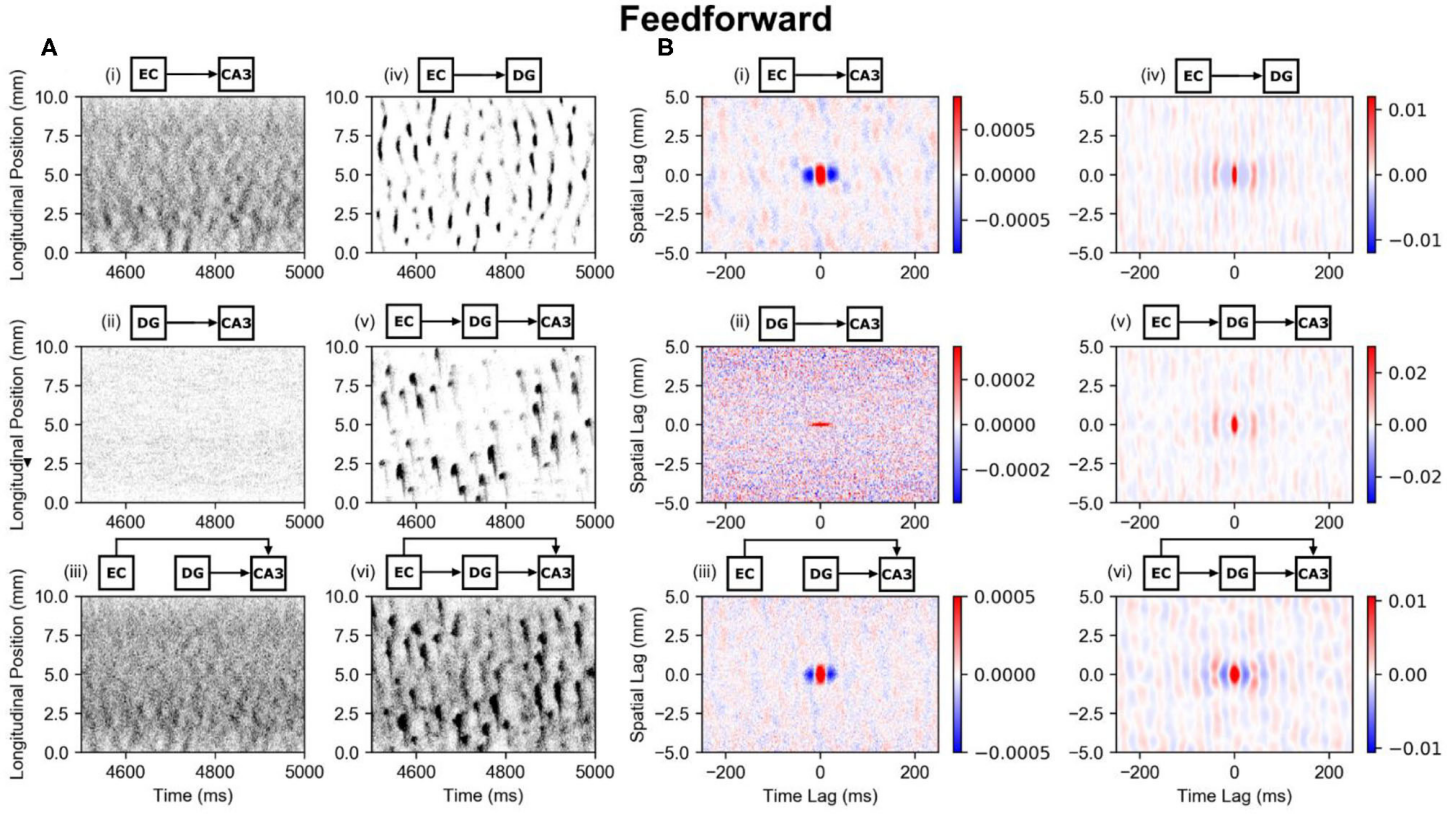
Raster plots and global correlation maps for the effects of the perforant path and mossy fibers on CA3 pyramidal cells. **(A)** Spiking activities of the CA3 pyramidal cells are indicated with black dots and are organized based on their longitudinal position and time of spike. The circuits above each subplot indicate which circuit configuration was used. All plots indicate CA3 pyramidal cell activity except for the EC-DG plot which shows dentate granule cell activity. **(B)** The corresponding space-time correlation maps are shown. The longitudinal-temporal cross-sections are shown at a transverse lag of 0 mm. Red areas represent positive correlation, and blue areas represent negative correlation. **(A,B)** (i) PP-CA3 model: CA3 response to random perforant path activity. (ii) rMF-CA3 model: CA3 response to random dentate granule cell activity. (iii) PP-rMF-CA3 model: CA3 response to both random perforant path and random dentate granule cell activity. (iv) PP-DG model: DG response to random perforant path activity. (v) wcMF-CA3 model: CA3 response to solely correlated dentate granule cell activity, i.e., CA3 response to disynaptic perforant activity via dentate granule cells. (vi) PP-wcMF-CA3 model: CA3 response due to random perforant path and correlated dentate granule cell activity.

There is a monosynaptic projection from entorhinal cortex to both dentate gyrus and CA3 meaning that the entorhinal cortex directly projects onto their principal neurons. Though the monosynaptic perforant path projection onto dentate gyrus has already been extensively covered (Hendrickson et al., 2016; Yu et al., 2018, 2019), a brief analysis is presented here for comparison with CA3. In CA3, the spatially and temporally uncorrelated entorhinal input (PP-CA3 model) was converted into network activity that exhibited a weak clustering (Figure 5A, i). Dentate granule cells (PP-DG model) responded with more visible clusters (Figure 5A, iv). A global space-time correlation map was computed with the CA3 pyramidal cells having a peak correlation of 0.0026 and the dentate granule cells having a peak correlation of 0.03 which is approximately an order of magnitude greater (Figure 5B, i,iv). The spatial extent of the correlation for both granule cells and CA3 pyramidal cells were nearly identical at approximately 1 mm.

These results indicate that the formation of clusters due to the perforant path are not unique to the dentate gyrus but can be generalized for different neural systems. Given a shared axonal distribution, the spatial extent of the correlation is preserved. However, the specific electrophysiology of the neuron types does affect the extent of temporal correlation. Other differences between the granule cells and CA3 pyramidal cells include the numbers of inputs that they receive from perforant path. The CA3 pyramidal cells receive much fewer inputs than granule cells and therefore share fewer inputs among their neighbors. This results in a lower peak correlation and noisier clusters.

#### Dentate Mossy Fiber Projection (rMF-CA3 and wcMF-CA3 Models)

The role of mossy fibers in organizing spatio-temporal activity was investigated within two conditions. The random mossy fiber (rMF) condition represented the dentate granule cell activity using an independent Poisson process that had the same mean firing rate as the weakly correlated mossy fiber (wcMF) condition which was 0.62 Hz. The wcMF represented the disynaptic propagation of entorhinal activity via mossy fibers to CA3, i.e., the dentate activity in Figure 5A, iv that was generated by the PP-DG model was used as the input to the CA3 pyramidal cells. The rMF created an input that was spatially and temporally uncorrelated. The wcMF created in an input with weak spatial and temporal correlation based on the dentate transformation of uncorrelated entorhinal activity. At the default synaptic parameters, the dentate activity was not sufficient to generate significant activity in the CA3. Therefore, the following analysis was performed with the synaptic weight increased by a factor of five.

The rMF resulted in CA3 activity that remained spatially uncorrelated and exhibited a weak temporal correlation with a peak of 0.001 (Figure 5A, ii). The wcMF resulted in the CA3 generating a spatio-temporal pattern that largely matched the spatio-temporal pattern of the dentate gyrus with a delay of 9 ms (Figure 5A, v). Additionally, the CA3 clusters included a “tail” that extended down toward the temporal pole and represents the downward turn that the mossy fiber trajectory follows after reaching the CA3a. The spatial structure of the CA3 correlation map remains largely similar to the dentate correlation map (Figure 5B, ii,v). These results indicate that in contrast to the perforant path projection, which organizes random activity into clusters, the mossy fibers do not imbue a spatial correlation to their postsynaptic population. Rather, the mossy fibers preserve the structure of the activity that is generated by the presynaptic population.

#### Combined Entorhinal and Dentate Projections (PP-rMF-CA3 and PP-wcMF-CA3 Models)

To explore the interactions between both the perforant path and mossy fiber projections, both rMF and wcMF were considered. In the PP-rMF-CA3 model, both the entorhinal cortex and dentate granule cell activity were randomly generated with an entorhinal mean firing rate of 5 Hz and a dentate mean firing rate of 0.62 Hz. These were both projected directly to the CA3. The PP-rMF-CA3 model eliminated the entorhinal projection to dentate. In the pPP-wcMF-CA3 model, the entorhinal cortex projected to both the dentate gyrus and CA3, and the CA3 received random input from entorhinal cortex and weakly correlated input from the dentate gyrus, which again represents the dentate gyrus' transformation of the random entorhinal input. These simulations were performed using the original synaptic weights, i.e., a scalar factor of one.

The PP-rMF-CA3 model resulted in the CA3 pyramidal cells generating a noisier version of the spatio-temporal pattern that was caused by the entorhinal projection by itself (Figure 5A, iii). This was expected as the random dentate input caused the CA3 to respond with spatially and temporally uncorrelated activity. The combination of these inputs results in the noisy pattern. The space-time correlation map supports this finding as the correlation structure is very similar to the correlation structure caused by the entorhinal projection but with a peak correlation that was roughly decreased by half (Figure 5B, iii).

The PP-wcMF-CA3 model caused the CA3 pyramidal cells to respond with a pattern that closely matched the dentate granule cell activity (Figure 5A, vi). Previously, the mossy fiber synaptic weights were multiplied by a factor of 5 to generate significant CA3 activity. Otherwise, the original mossy fiber synaptic weight generated almost no CA3 activity. In combination with the perforant path, the mossy fibers at their original strength were able to propagate the dentate clusters and cause similar clusters within CA3. The CA3 clusters were noisier than the dentate clusters (Figure 5A, iv), and it is likely that the CA3 pattern is some combination of the patterns caused by the entorhinal and dentate activity individually. However, the perforant path projection was able to nonlinearly interact with the mossy fibers to markedly reinforce the dentate input pattern. The combination of these two systems may serve to enhance and propagate the patterns generated by the dentate granule cells.

#### Associational System (PP-wcMF-A-CA3 Model)

The perforant path and mossy fiber inputs together resulted in the preservation of the dentate pattern within the CA3, at least along the longitudinal axis. However, the CA3 contains an extremely strong associational system which could alter the pattern due to the excitatory feedback that the associational system provides. The subsequent studies explored the further transformation of spatio-temporal pattern that resulted when the associational system was added (the PP-wcMF-A-CA3 model). Separate simulations were run with the synaptic weight of the associational system set to 0.1, 0.2, 0.5, 1, 10, and 100% of the original value.

**Figure 6:**
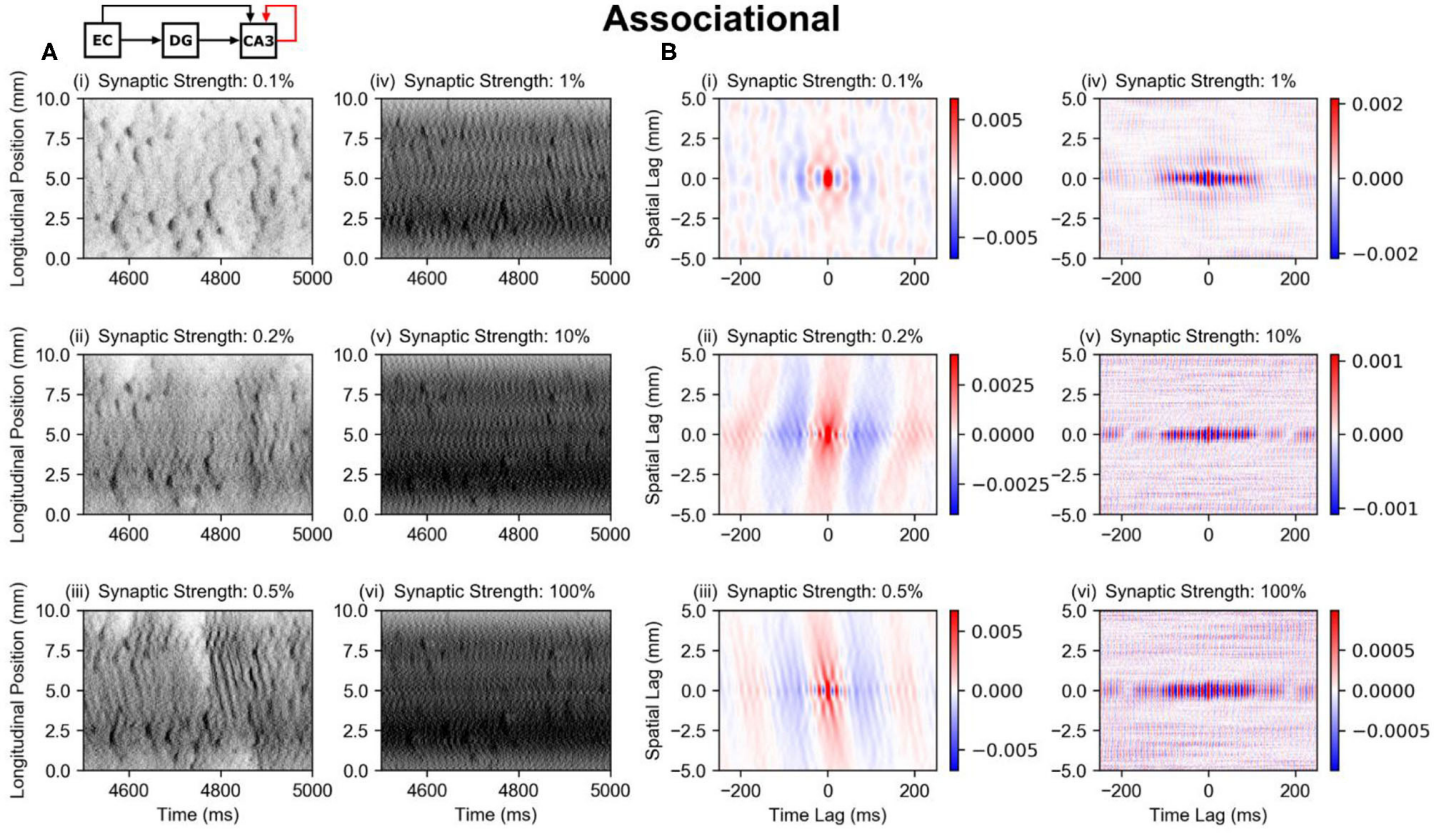
Raster plots and global correlation maps for the results using the PP-wcMF-A-CA3 model, which includes the perforant path, mossy fiber, and associational projections to CA3. In the subplots, the strength of the associational synapses was modulated as indicated by the subplot titles. All other synapses remained at their experimentally constrained strength. **(A)** Raster plots of the CA3 pyramidal cells. **(B)** Longitudinal-temporal cross-sections of the correlation map at a transverse lag of 0 mm. Red areas represent positive correlation, and blue areas represent negative correlation.

The strength of the associational system can be predominantly attributed to the number of inputs that a CA3 pyramidal cell receives from other CA3 pyramidal cells rather than the strength of the individual EPSPs. This number lies within the tens of thousands which is at least one order of magnitude larger than the numbers of inputs received from other afferents including the perforant path and mossy fibers. The average firing rate of the simulated CA3 pyramidal cells started at 11 Hz with no associational system and increased nonmonotonically toward 70 Hz with increasing synaptic strength (Supplementary Figure 2A), indicating that the CA3 activity was much less sparse than the DG which exhibited an average firing rate of 0.62 Hz. Furthermore, the spatio-temporal patterns appear to change little at 1, 10, and 100% of the full strength (Figure 6A, iv–vi), i.e., the spatio-temporal pattern did not change substantially. In spatio-temporal pattern and firing rate, the CA3 approached a particular state asymptotically, which indicated that the CA3 was approaching a saturated state, i.e., the system was behaving nonlinearly. It is only at 0.1–0.5% of the original synaptic weight (Figure 6A, i–iii) that the associational system appears to significantly affect the spatio-temporal pattern. At these synaptic weight levels, the transformation of the clusters can be observed. The diffuse axonal distributions of the CA3 pyramidal cells appears to expand the spatial size of the clusters. This is also demonstrated by the correlation maps which show the increase in the extent of the spatial correlation with the increase in synaptic weight (Figure 6B).

Regardless of the strength of the associational system, the original clusters appear to continue to persist at all synaptic weights. The associational only serves to modify them and add inter-cluster noise or oscillation. This is notable because the peak pairwise correlation of the dentate granule cells is very low at 0.02, and the average firing rate is also very sparse at 0.62 Hz. Despite the low correlation and sparse firing conditions, the dentate gyrus causes clusters in CA3 pyramidal cells that remain even when the associational system is at full strength.

### Longitudinal-Transverse Cross-Sections of Global Correlation Maps

Previously, the visualization of activity and correlation was limited to a single spatial dimension, the lag along the longitudinal axis. However, the CA3 exhibits a transverse organization that is hidden when only considering the longitudinal extent. The longitudinal-temporal view of the correlation maps demonstrated their ability to capture the basic structure of the features observed in the raster plots, i.e., the basic cluster shape. The three-dimensional correlation maps that were computed also incorporate the transverse relation to correlated activity. Here, we present the longitudinal-transverse cross-sections of the correlation maps at different time lags to reveal the temporal evolution of the two-dimensional spatial structure of correlation. The color maps were thresholded to 30% of the maximum value to emphasize the contributions of the weaker negative correlations that were present.

#### Perforant Path and Mossy Fibers

**Figure 7:**
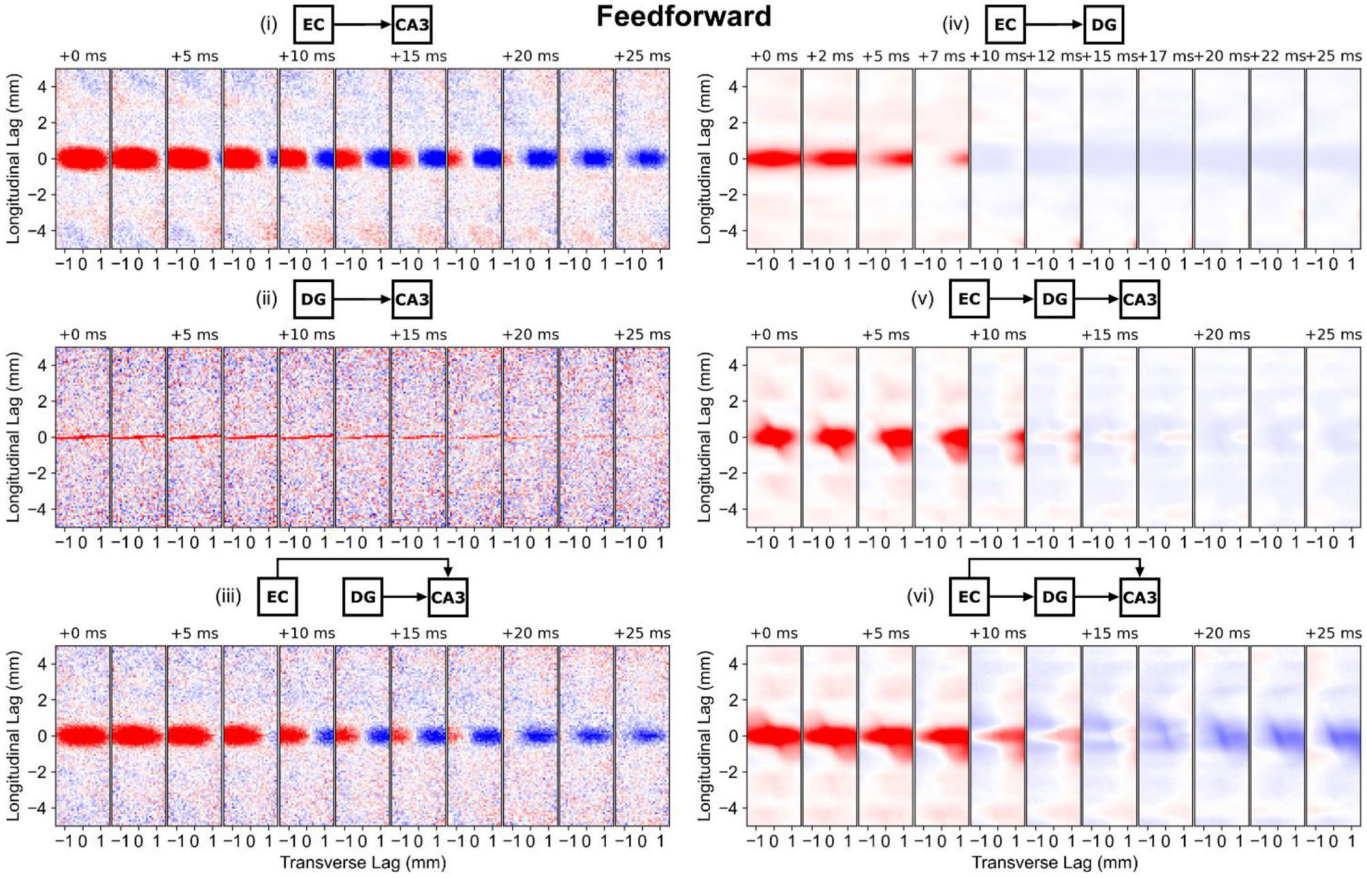
Longitudinal-transverse cross-sections of global correlation maps at different temporal lags for simulations involving the perforant path and mossy fibers. The evolution of spatial correlation across positive time lags are shown. Red areas represent positive correlation, and blue areas represent negative correlation. (i) PP-CA3 model: CA3 response to random perforant path activity. (ii) rMF-CA3 model: CA3 response to random dentate granule cell activity. (iii) PP-rMF-CA3 model: CA3 response to both random perforant path and random dentate granule cell activity. (iv) PP-DG model: DG response to random perforant path activity. (v) wcMF-CA3 model: CA3 response to solely correlated dentate granule cell activity, i.e., CA3 response to disynaptic perforant activity via dentate granule cells. (vi) PP-wcMF-CA3 model: CA3 response due to random perforant path and correlated dentate granule cell activity.

The longitudinal-transverse views of correlation demonstrate that the spatial structure is predominantly dependent on the methods used to represent the axonal anatomy. For the entorhinal projections which were represented using a gaussian (the PP-CA3 and PP-DG models), the spatial correlation maintains an elliptical shape with a longitudinal span that matches the standard deviation of the gaussian (Figure 7, i,iv). The differences in the temporal dynamics for the entorhinal effect on dentate granule cells and CA3 pyramidal cells may be due to differences in biophysics, electrophysiology, and number of inputs received by the respective populations. In the CA3, a positive and negative correlation moves across the transverse axis which represents the transverse propagation of entorhinal input from the CA3c to the CA3a.

With only mossy fiber input to the CA3, the random condition (rMF-CA3 model) resulted in a horizontal stripe spatial correlation that spanned the transverse axis which represents the thin nature of the mossy fibers (Figure 7, ii). The spatial correlation faded with time. The weakly correlated condition (wcMF-CA3 model) largely preserved the correlation structure of the dentate granule cells with the addition of a diagonal element which represented the downward turn of the mossy fibers in the CA3a (Figure 7, iv,v). As the temporal lag progressed, the shape of the correlation changed before moving into a weakly negative phase. With the combination of both perforant path and mossy fiber afferents, the random condition (the PP-rMF-CA3 model) again exhibited a correlation structure that was similar to the entorhinal case, but the extent of the spatial correlation began to shrink in the negative phase (Figure 7, iii). In the weakly correlated condition (the PP-wcMF-CA3 model), both the perforant path and mossy fiber related correlation structures appeared superimposed with the diagonal stripe appearing and a stronger negative phase (Figure 7, vi).

The main observation is that the spatial correlation was nonmonotonic and was not static in time, i.e., spatial correlation was dynamic. The spatial correlation can travel based on the direction of propagation and does not merely oscillate between positive and negative in a fixed position. Furthermore, the shape of the correlation changes over time. The correlation is both displaced and morphed partly due to direction of propagation and the interactions between different afferents.

#### Associational System

**Figure 8:**
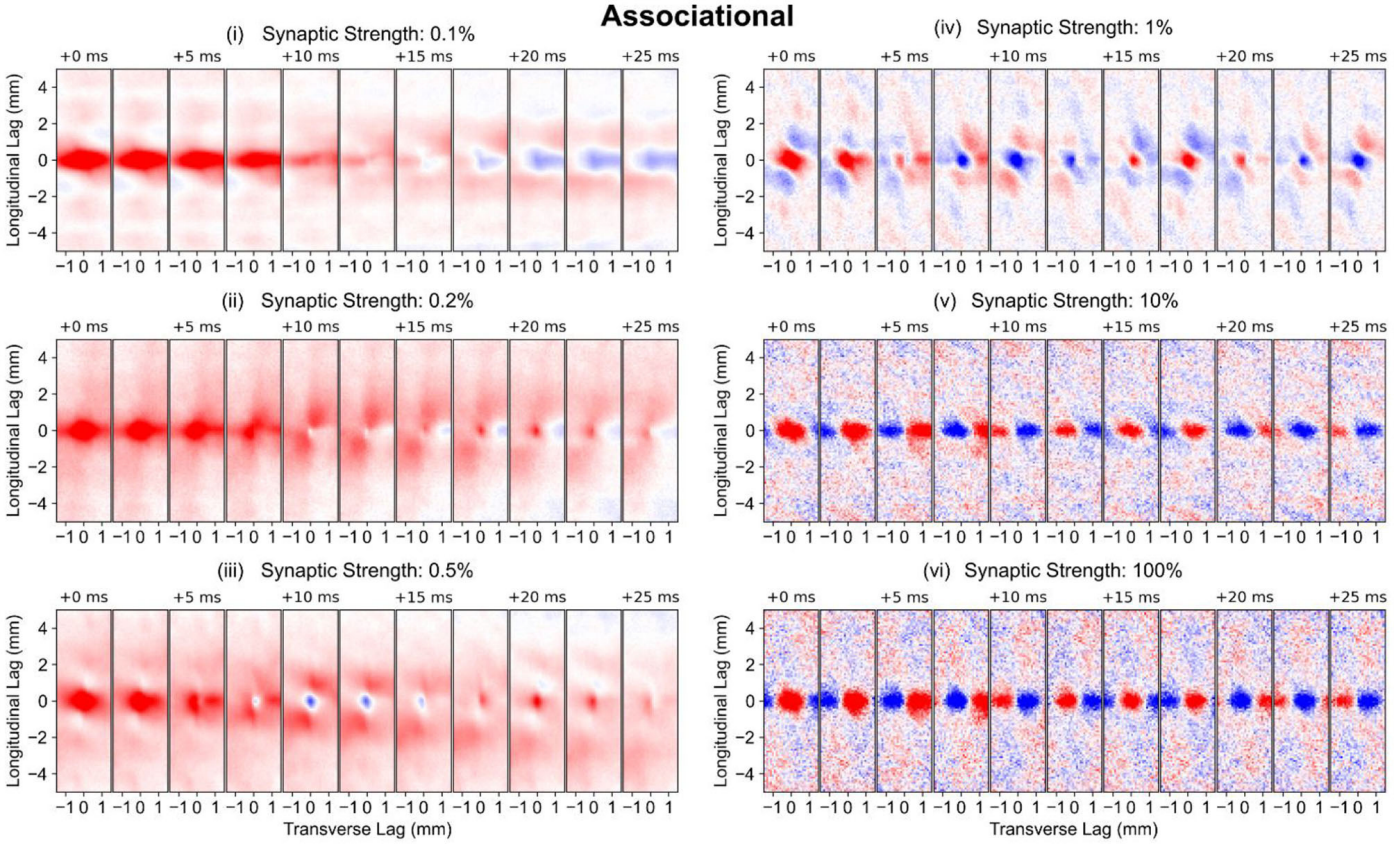
Longitudinal-transverse cross-sections of global correlation maps at different temporal lags for simulations using the PP-wcMF-A-CA3 model, which includes the perforant path, mossy fiber, and associational projections to CA3. In the subplots, the strength of the associational synapses was modulated as indicated by the subplot titles. All other synapses remained at their experimentally constrained strength. The evolution of spatial correlation across positive time lags are shown. Red areas represent positive correlation, and blue areas represent negative correlation.

The longitudinal-transverse view of the correlation reveals the role of the associational system (PP-wcMF-A-CA3 model) in increasing the spatial extent of the correlation (Figure 8). The spatial extent of correlation increased with synaptic weights from 0.1–0.5% strength to encompass almost the entire CA3 extent (Figure 8, i–iii), though the strongest correlation was still localized to the same area that was caused by the entorhinal projection. As the strength of the synaptic weight was increased to 1–100%, the extent of spatial correlation became reduced to an area that was smaller than the correlation caused by the entorhinal cortex (Figure 8, iv–vi). At 1% strength, a spatial correlation pattern consisting of a positive region surrounded by negative correlation emerged. The polarity of this pattern switched between positive-negative and negative-positive over time. At 10 and 100% strength, the spatial correlation and its temporal evolution appeared almost identical. A repeating pattern of positive and negative correlation moves along the transverse axis over time. These results verify that the extensive axon distribution of the CA3 pyramidal cells does increase the area of spatial correlation during the low synaptic strengths. At higher synaptic strengths, the correlation oscillated between positive and negative which is consistent with the highly oscillatory nature of the spiking activity. At 1% strength, a unique pattern of positive and negative correlation emerged (Figure 8, iv).

### Local Correlation Maps

One issue with the global correlation maps computed previously is that the anatomy of the various projection changes depending on the location within the CA3. In particular, the trajectory of the mossy fiber changes along the transverse axis, and the CA3 pyramidal cell axons change substantially depending on their origin on both the longitudinal and transverse axes. While the global maps used every neuron pair in its computation of correlation, local maps were created by only consider neuron pairs in which at least one neuron of the pair was located in a particular area within the CA3. The CA3 was divided into nine sections. Along the longitudinal axis, three overlapping windows were defined which were centered at 7.5, 5.0, and 2.5 mm that extended 2.5 mm above and below the midpoint. Along the transverse axis, the windows were restricted to the CA3c, CA3b, and CA3a. This restriction allowed the correlation maps for a local region in space within the CA3 to be computed, in contrast to the global map which averages across every neuron (Figure 4).

**Figure 9:**
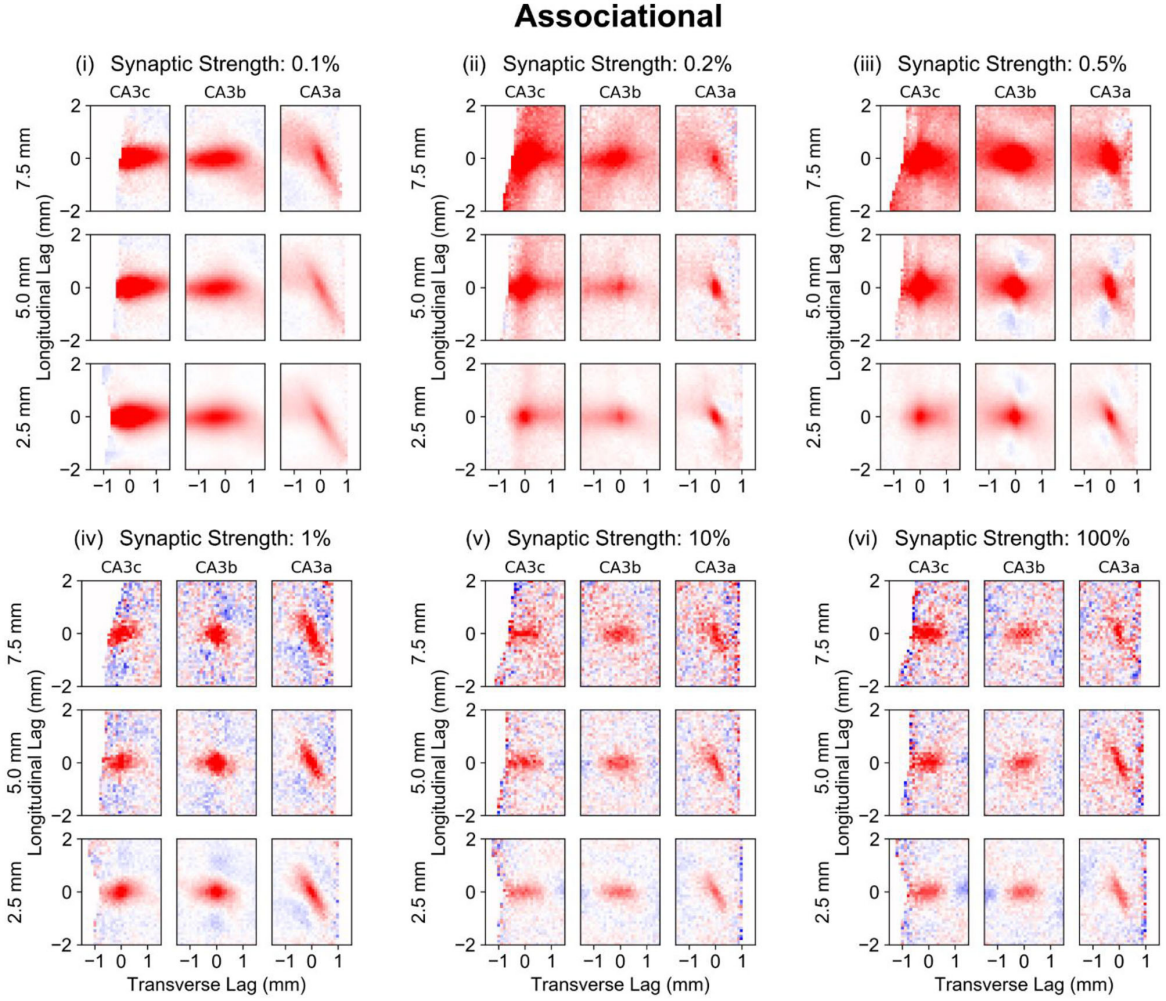
Local correlation maps for simulations using the PP-wcMF-A-CA3 model, which includes the perforant path, mossy fiber, and associational projections to CA3. In the subplots, the strength of the associational synapses was modulated as indicated by the subplot titles. All other synapses remained at their experimentally constrained strength. Longitudinal-transverse cross-sections at a temporal lag of 0 ms are shown. Nine local correlation maps were computed for each synaptic strength which represents the local spatio-temporal correlation based on the longitudinal-transverse region as divided into CA3c, CA3b, and CA3a along the transverse axis (left to right) and roughly into the septal, middle, and temporal sections (top to bottom) along the longitudinal axis. The longitudinal boundaries were defined with a window size of 5 mm and centered at 7.5 (septal), 5.0, and 2.5 (temporal) mm along the longitudinal axis. Red areas represent positive correlation, and blue areas represent negative correlation.

Considering the local correlation maps when the associational system was included (PP-wcMF-A-CA3 model), the influence of the mossy fiber trajectory on the correlation structure along the transverse axis can be seen when then associational synaptic weight was reduced to 0.1% (Figure 9, i). Within the CA3c and CA3b, the correlation is predominantly horizontal while the correlation becomes diagonal within CA3a. This again highlights the influence of axonal anatomy on the correlation structure. It also reveals that the diagonally-organized positive/negative correlations seen in the global maps were due to the averaging of the CA3a correlation structure with the correlations from CA3b and CA3c. The local correlation maps were able to identify and separate the contributions of the different CA3 divisions (Figure 9, i) toward the global correlation map (Figure 7, vi).

At 0.02 and 0.05% (Figure 9, ii,iii), the differences in correlation structure among the different CA3 sections due to the CA3 axonal anatomy become more apparent. The magnitude and spatial extent of correlation is largest within the septal CA3c which becomes smaller toward the temporal CA3a. As the synaptic weights approach 100% strength, the correlations become confined to a smaller area.

### Influence of Projections on Peak Correlation

**Figure 10:**
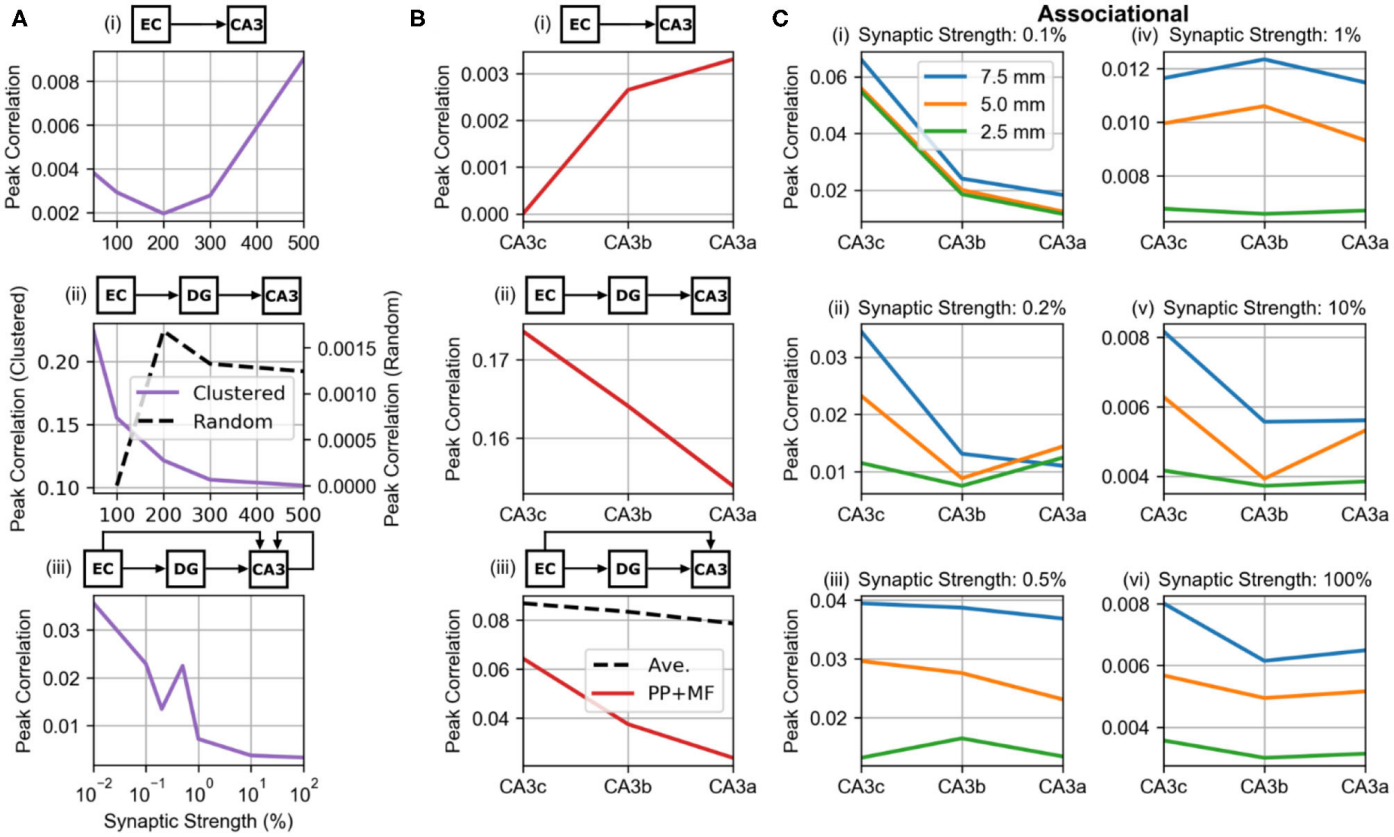
Relations between peak correlation and synaptic strength and location. **(A)** The effect of synaptic strength on peak correlation from the global maps. The middle row plot shows the relation under the clustered (solid purple line) and random (dashed black line) conditions of dentate input. (**A**, i) PP-CA3 model: CA3 response to random perforant path activity. (**A**, ii) wcMF-CA3 model: CA3 response to solely correlated dentate granule cell activity, i.e., CA3 response to disynaptic perforant activity via dentate granule cells. (**A**, iii) PP-wcMF-A-CA3 model: CA3 response due to random perforant path and correlated dentate granule cell activity in the presence of the associational system. **(B)** The differences in peak correlation based on transverse position from the global maps. In the bottom plot, the effect of the combined perforant path and mossy fiber input (solid red line) and the average of the individual effects of the two pathways (dashed black line) are shown. (**B**, i) PP-CA3 model: CA3 response to random perforant path activity. (**B**, ii) wcMF-CA3 model: CA3 response to solely correlated dentate granule cell activity, i.e., CA3 response to disynaptic perforant activity via dentate granule cells. (**B**, iii) PP-wcMF-CA3 model: CA3 response due to random perforant path and correlated dentate granule cell activity without the associational system. **(C)** The variation in peak correlation from the local maps as a function of longitudinal and transverse position are shown from simulations using the PP-wcMF-A-CA3 model, which includes the perforant path, mossy fiber, and associational projections to CA3. The strength of the associational system is modulated as indicated by the subplot titles.

The peak correlations were plotted as a function of synaptic strength based on the global maps to investigate how the synaptic strength of the different projections affected maximum correlation (Figure 10A). For the perforant path and mossy fibers, the synaptic strength was varied to be 50, 200, 300, 400, and 500% of the original value. The synaptic weight of the perforant path (PP-CA3 model) had a nonlinear relation to the peak correlation which initially decreased until twice the original strength and then continued to increase (Figure 10A, i). In general, however, the peak correlation due to the entorhinal projection to CA3 was very weak and stayed below 0.01. Under the weakly correlated condition (wcMF-CA3 model), the mossy fibers generated a monotonic relation between CA3 peak correlation and synaptic strength that decreased toward an asymptotic value of 0.1 (Figure 10A, ii). However, the random condition (rMF-CA3 model) had an opposite and nonmonotonic effect, and its peak correlation was three orders of magnitude lower than for the weakly correlated condition (Figure 10A, ii). The associational system (PP-wcMF-A-CA3 model) caused the peak correlation to nonmonotonically decrease toward an asymptotic value of 0.003 as the synaptic strength increased (Figure 10A, iii). Though generally decreasing, the correlation unexpectedly increased at an associational strength of 0.05% before continuing to decrease.

Using the local correlation maps, the distribution of correlation along the transverse extent was evaluated for the feedforward projections (Figure 10B). The correlation caused by the perforant path (PP-CA3 model) increased from CA3c to CA3a which is explained by the increase in synaptic density along the transverse axis (Figure 10B, i). For the wcMF-CA3 model, correlations decreased from CA3c to CA3a (Figure 10B, ii). This is due to the decrease in density of synapses along the transverse axis. When both the perforant path and mossy fibers were connected (PP-wcMF-CA3 model), the correlation due to the mossy fibers dominated resulting in a decrease in correlation from CA3c to CA3a (Figure 10B, iii). However, the correlation was not simply an average between the correlations from the individual projections. The combined effect was lower than what an average would predict (Figure 10B, iii).

Including the associational system (PP-wcMF-A-CA3 model), the trend in peak correlation along both the longitudinal and transverse axes were measured (Figure 10C). In general, peak correlation decreased from the dorsal/septal pole to the ventral/temporal pole and decreased from CA3c to CA3a.

## Discussion

In this study, anatomical data was used to constrain a spatially-dependent connectivity for the excitatory projections to and within the CA3 subfield of hippocampus including the perforant path, mossy fibers, and associational system. The present work represents an extensively detailed connectivity for the entorhinal-dentate-CA3 network concerning the major excitatory afferents to the dentate gyrus and CA3 and represents connectivity at the microscale and mesoscale levels. A major outcome in constraining the connectivity was to include the variations in the spatial distribution of axons along both the longitudinal and transverse axes. These axes are sufficient to represent the full three-dimensional structure of the hippocampus. Theoretical studies that incorporated two spatial dimensions had constructed radially symmetric connectivity structures that could easily be analyzed along a single radial dimension (Rosenbaum et al., 2016; Senk et al., 2018; Huang et al., 2019). These studies with symmetric connectivity are able to generate rich sets of dynamics and lay the foundation for studying two-dimensional networks. However, the axonal distributions in hippocampus are far from symmetric. Furthermore, the variations in CA3 properties, e.g., connectivity and dendritic morphology, within its transverse axis are well-documented and represent important features that cannot be ignored. Incorporating the details relevant to both spatial axes resulted in the emergence of a heterogeneous spatial correlation structure, which has implications toward a topographic organization of information encoding along both the longitudinal and transverse axes. Additionally, the correlations were nonmonotonic in that they would not strictly decrease, and the correlations were dynamic with changes in their spatial structure at different time lags.

### Perforant Path Generates Lower Correlation in CA3 Than Dentate Gyrus

Also using the entorhinal-dentate network, a relation between the size of the axon terminal field and the resulting correlation was revealed in Hendrickson et al. (2016) and Yu et al. (2018). The size of the axon terminal field and the size of the clusters in the population activity were linearly related. However, the entorhinal-dentate projection is relatively dense compared to the entorhinal-CA3 projection as dentate granule cells receive approximately 3,000 entorhinal inputs compared to CA3 pyramidal cells which can receive between 0 and 1,658 entorhinal inputs (Table 1). The present study revealed that the perforant path still generates clusters within CA3, though the correlation is much lower which resulted in noisier clusters (Figure 5A).

### Mossy Fibers Propagate Correlation Structure From Dentate Gyrus to CA3

A larger unknown was the role of mossy fibers in the propagation of correlation. The mossy fibers of dentate granule cells represented the smallest extreme in size as they are fibers rather than fields. Though the number of mossy fiber inputs that CA3 pyramidal cells receive is extremely low with a mode of 38 (Supplementary Figure 1), the size of the EPSP is 3.2 mV which is over ten times greater than the EPSPs caused by the perforant path at 0.2 mV. The analysis of the longitudinal correlation indicated that the mossy fibers contribute little to the spatial correlation structure beyond that is already present within dentate granule cells. In other words, mossy fibers well-preserve and propagate the correlation structure that its presynaptic population, i.e., dentate granule cells, already expresses. However, the longitudinal-transverse analysis of the random condition shows that mossy fibers do contribute to a spatial correlation along the transverse axis, albeit a practically negligible correlation at <0.0001.

The combination of the perforant path and mossy fibers was shown to enhance the pattern carried in the mossy fibers. The mossy fibers alone did not generate significant activity within CA3. Experimental studies support that mossy fibers do not reliably cause action potentials at low frequencies, which is the case in these simulations (Urban et al., 2001). However, when the perforant path was added, the dentate patterns of activity were reinforced and perpetuated in the CA3 activity (Figure 5A). The enhancement of the mossy fiber pattern supports the view of mossy fibers as conditional detonators which may need concurrent activation of multiple synapses in order to elicit a spike in the pyramidal cells, e.g., mossy fiber, perforant path, associational (Henze et al., 2002).

### Associational System Preserves and Modulates Mossy Fiber Induced Patterns

However, it was not known how much of the dentate pattern would persist within CA3 after the associational system was included. The effect of a recurrent excitatory circuit in neural systems has commonly been shown to generate highly synchronized and oscillatory behavior (Le Duigou et al., 2014; Hendrickson et al., 2015). This was observed in the CA3 associational system due to the highly dense nature of the projection in which a CA3 pyramidal cell can receive between 18,000 and 29,000 inputs from other CA3 pyramidal cells. With synaptic weights at 0.1–0.5% of the original strength, the CA3 did not enter a synchronous, oscillatory state (Figure 6A), and an increase in the size of spatial correlation was observed (Figures 8, 9). This indicates that weak excitatory recurrent circuits can expand the extent of spatial correlation. One of the interesting findings in the associational results was that the dentate-based clusters persisted within all synaptic weight values, i.e., even within the synchronized, oscillatory state. This finding further supports that the dentate activity acts as a major driver of the spatio-temporal patterns generated by the CA3.

### Correlation and Functional Gradients Along Both Longitudinal and Transverse Axes Within CA3

The topographic organization of connectivity resulted in heterogeneous correlation structures that varied along the longitudinal and transverse axes within the CA3. First, there was a clear trend toward higher correlations in the dorsal/septal region of the CA3 vs. ventral/temporal region. Second, correlations were higher in the proximal/CA3c region vs. the distal/CA3a region. These ultimately combine to indicate that peak correlation decreases from dorsal-proximal CA3 to the ventral-distal CA3. Additionally, the spatial extent of correlation followed the same gradient with a larger size of spatial correlation dorsally/proximally and a smaller size of spatial correlation ventrally/distally.

These correlation gradients may functionally indicate the extent to which information is integrated by a CA3 pyramidal cell. With larger sizes in correlation, a CA3 pyramidal cell is integrating information across more neurons, which may result in a CA3 organization with more “general” neurons dorsally/proximally and more specialized neurons ventrally/distally. Another interpretation is that neuronal activity may be more similar to one another in the dorsal/proximal region vs. ventral/distal region. Experimental work had discovered that a positive relation between pairwise spike correlation and overlap of place fields within CA3 and CA1 (Hampson et al., 1996). Under the theory of pattern separation and pattern completion (Yassa and Stark, 2011), this suggests that pattern completion as a population may be stronger where correlation between neurons is higher.

A longitudinal gradient in function has been reported experimentally (Small, 2002; Strange et al., 2014; Papaleonidopoulos et al., 2017), and such a relation between axonal anatomy and the encoding of spatial information had been previously explored with the same entorhinal-dentate network used in the present study (Yu et al., 2019). A transverse gradient has also been reported within CA3 with respect to pattern completion (Lee et al., 2015).

### Validation

As a platform for investigating the system properties of hippocampus, it is important to validate the model at higher levels, e.g., population and network levels. Lower level validation is already performed in constraining the parameters for neuron electrophysiology and synaptic conductance waveforms. Some higher-level validation has been performed using place fields and spatial information (Yu et al., 2019), and local field potential generation (Bingham et al., 2018). The present CA3 network lacks inhibition and is comparable to a CA3 for which a GABA_A_ blocker has been applied. One experimental study observed that the power of CA3 population oscillations at 210 Hz increased with the application of the GABA_A_ blocker bicuculline. This increase is also observed with the simulations (Supplementary Figure 2C). Pairwise spike correlation values that have been reported in experimental studies are vary between 0.005 and 0.025 (Hampson et al., 1996; Dombeck et al., 2010). The peak correlation values from the simulations are well within these ranges. Furthermore, other studies have reported a general decay in pairwise correlation as a function of distance between neuron pairs (Hirase et al., 2001; Dombeck et al., 2010). This relation is present in other cortical areas as well (Rosenbaum et al., 2016; Safavi et al., 2018) which supports the notion that such correlations may be present in other brain areas.

### Future Work

The CA3 network in the study excluded any forms of extrinsic inhibition due to interneurons as the role of the afferent excitatory projections were not yet known. However, the present results establish the groundwork upon which the contributions of the various interneuron types in further transforming the spatio-temporal patterns of spiking and correlation can be investigated.

Additionally, the simulations used an input paradigm designed to contain zero spatial or temporal correlations to reveal how the anatomically-constrained connectivity of the network may imbue the population activity with correlation. Though the input firing rates may represent a resting state type of network, the input was not physiologically. Later work will aim to behaviorally-driven input such as the grid cells in the medial entorhinal cortex to investigate how physiologically-relevant correlation in the input may be processed by CA3, and grid cell input had previously been used to investigate the entorhinal-dentate network version of the model (Yu et al., 2019).

## Data Availability Statement

The code used to perform the simulations can be found at the following link: https://github.com/genejongyu/reducedCA3network.git. For additional data, inquiries can be directed to the corresponding author.

## Author Contributions

GY developed the network model, designed and performed the analysis, and prepared the manuscript. J-MB assisted in the preparation of the manuscript. TB oversaw the project scope, project conception, and worked to maintain funding to support the project. All authors contributed to the article and approved the submitted version.

## Conflict of Interest

The authors declare that the research was conducted in the absence of any commercial or financial relationships that could be construed as a potential conflict of interest.

## References

[B1] Acsády L. Kamondi A. Sik A. Freund T. Buzsáki G. (1998). GABAergic Cells are the major postsynaptic targets of mossy fibers in the rat hippocampus. J. Neurosci. 18, 3386–3403. 10.1523/JNEUROSCI.18-09-03386.1998 9547246 PMC6792657

[B2] Andersen P. (1975). “Organization of hippocampal neurons and their interconnections,” in The Hippocampus, eds R. L. Isaacson and K. H. Pribram (Boston, MA: Springer), 155-175.

[B3] Andersen P. Soleng A. F. Raastad M. (2000). The hippocampal lamella hypothesis revisited. Brain Res. 886, 165–171. 10.1016/S0006-8993(00)02991-7 11119694

[B4] Billeh Y. N. Cai B. Gratiy S. L. Dai K. Iyer R. Gouwens N. W.. (2020). Systematic integration of structural and functional data into multi-scale models of mouse primary visual cortex. Neuron 106, 388–403.e18. 10.1016/j.neuron.2020.01.040 32142648

[B5] Bingham C. S. Loizos K. Yu G. J. Gilbert A. Bouteiller J.-M. C. Song D.. (2018). Model-based analysis of electrode placement and pulse amplitude for hippocampal stimulation. IEEE Trans. Biomed. Eng. 65, 2278–2289. 10.1109/TBME.2018.2791860 29993519 PMC6224291

[B6] Chan H. K. Yang D.-P. Zhou C. Nowotny T. (2016). Burst firing enhances neural output correlation. Front. Comput. Neurosci. 10:42. 10.3389/fncom.2016.00042 27242499 PMC4860405

[B7] Darshan R. van Vreeswijk C. Hansel D. (2018). Strength of correlations in strongly recurrent neuronal networks. Phys. Rev. X 8:031072 10.1103/PhysRevX.8.031072

[B8] Dettner A. Münzberg S. Tchumatchenko T. (2016). Temporal pairwise spike correlations fully capture single-neuron information. Nat. Commun. 7:13805. 10.1038/ncomms13805 27976717 PMC5171810

[B9] Dolorfo C. L. Amaral D. G. (1998). Entorhinal cortex of the rat: topographic organization of the cells of origin of the perforant path projection to the dentate gyrus. J. Comp. Neurol. 398, 25–48. 10.1002/(SICI)1096-9861(19980817)398:1<25::AID-CNE3>3.0.CO;2-B 9703026

[B10] Dombeck D. A. Harvey C. D. Tian L. Looger L. L. Tank D. W. (2010). Functional imaging of hippocampal place cells at cellular resolution during virtual navigation. Nat. Neurosci. 13, 1433–1440. 10.1038/nn.2648 20890294 PMC2967725

[B11] Halliday D. M. (2000). Weak, stochastic temporal correlation of large-scale synaptic input is a major determinant of neuronal bandwidth. Neural Comput. 12, 693–707. 10.1162/089976600300015754 10769327

[B12] Hampson R. E. Byrd D. R. Konstantopoulos J. K. Bunn T. Deadwyler S. A. (1996). Hippocampal place fields: relationship between degree of field overlap and cross-correlations within ensembles of hippocampal neurons. Hippocampus 6, 281–293. 10.1002/(SICI)1098-1063(1996)6:3<281::AID-HIPO6>3.0.CO;2-Q 8841827

[B13] Helias M. Tetzlaff T. Diesmann M. (2014). The correlation structure of local neuronal networks intrinsically results from recurrent dynamics. PLoS Comput. Biol. 10:e1003428. 10.1371/journal.pcbi.1003428 24453955 PMC3894226

[B14] Hemond P. Epstein D. Boley A. Migliore M. Ascoli G. A. Jaffe D. B. (2008). Distinct classes of pyramidal cells exhibit mutually exclusive firing patterns in hippocampal area CA3b. Hippocampus 18, 411–424. 10.1002/hipo.20404 18189311 PMC4339291

[B15] Hendrickson P. J. Yu G. J. Song D. Berger T. W. (2015). Interactions between inhibitory interneurons and excitatory associational circuitry in determining spatio-temporal dynamics of hippocampal dentate granule cells: a large-scale computational study. Front. Syst. Neurosci. 9:155. 10.3389/fnsys.2015.00155 26635545 PMC4647071

[B16] Hendrickson P. J. Yu G. J. Song D. Berger T. W. (2016). A million-plus neuron model of the hippocampal dentate gyrus: critical role for topography in determining spatiotemporal network dynamics. IEEE Trans. Biomed. Eng. 63, 199–209. 10.1109/TBME.2015.2445771 26087482 PMC4745257

[B17] Henze D. A. Wittner L. Buzsáki G. (2002). Single granule cells reliably discharge targets in the hippocampal CA3 network in vivo. Nat. Neurosci. 5, 790–795. 10.1038/nn887 12118256

[B18] Hirase H. Leinekugel X. Csicsvari J. Czurkó A. Buzsáki G. (2001). Behavior-dependent states of the hippocampal network affect functional clustering of neurons. J. Neurosci. 21, RC145–RC145. 10.1523/JNEUROSCI.21-10-j0003.2001 11319243 PMC6762491

[B19] Hong S. Ratte S. Prescott S. A. De Schutter E. (2012). Single neuron firing properties impact correlation-based population coding. J. Neurosci. 32, 1413–1428. 10.1523/JNEUROSCI.3735-11.2012 22279226 PMC3571732

[B20] Huang C. Ruff D. A. Pyle R. Rosenbaum R. Cohen M. R. Doiron B. (2019). Circuit models of low-dimensional shared variability in cortical networks. Neuron 101, 337–348.e4. 10.1016/j.neuron.2018.11.034 30581012 PMC8238668

[B21] Ishizuka N. Cowan W. M. Amaral D. G. (1995). A quantitative analysis of the dendritic organization of pyramidal cells in the rat hippocampus. J. Comp. Neurol. 362, 17–45. 10.1002/cne.903620103 8576427

[B22] Ishizuka N. Weber J. Amaral D. G. (1990). Organization of intrahippocampal projections originating from CA3 pyramidal cells in the rat. J. Comp. Neurol. 295, 580–623. 10.1002/cne.902950407 2358523

[B23] Jahr C. Stevens C. (1990). Voltage dependence of NMDA-activated macroscopic conductances predicted by single-channel kinetics. J. Neurosci. 10, 3178–3182. 10.1523/JNEUROSCI.10-09-03178.1990 1697902 PMC6570236

[B24] Kleppe I. C. Robinson H. P. C. (1999). Determining the activation time course of synaptic AMPA receptors from openings of colocalized NMDA receptors. Biophys. J. 77, 1418–1427. 10.1016/S0006-3495(99)76990-0 10465753 PMC1300430

[B25] Kress G. J. Dowling M. J. Meeks J. P. Mennerick S. (2008). High threshold, proximal initiation, and slow conduction velocity of action potentials in dentate granule neuron mossy fibers. J. Neurophysiol. 100, 281–291. 10.1152/jn.90295.2008 18480368 PMC2493481

[B26] Kriener B. Helias M. Aertsen A. Rotter S. (2009). Correlations in spiking neuronal networks with distance dependent connections. J. Comput. Neurosci. 27, 177–200. 10.1007/s10827-008-0135-1 19568923 PMC2731936

[B27] Kumar A. Rotter S. Aertsen A. (2010). Spiking activity propagation in neuronal networks: reconciling different perspectives on neural coding. Nat. Rev. Neurosci. 11, 615–627. 10.1038/nrn2886 20725095

[B28] Lawrence J. J. Grinspan Z. M. McBain C. J. (2003). Quantal transmission at mossy fibre targets in the CA3 region of the rat hippocampus. J. Physiol. 554, 175–193. 10.1113/jphysiol.2003.049551 14678500 PMC1664753

[B29] Le Duigou C. Simonnet J. Teleñczuk M. T. Fricker D. Miles R. (2014). Recurrent synapses and circuits in the CA3 region of the hippocampus: an associative network. Front. Cell. Neurosci. 7:262. 10.3389/fncel.2013.00262 24409118 PMC3884140

[B30] Lee H. Wang C. Deshmukh S. S. Knierim J. J. (2015). Neural population evidence of functional heterogeneity along the CA3 transverse axis: pattern completion versus pattern separation. Neuron 87, 1093–1105. 10.1016/j.neuron.2015.07.012 26298276 PMC4548827

[B31] Marasco A. Limongiello A. Migliore M. (2012). Fast and accurate low-dimensional reduction of biophysically detailed neuron models. Sci. Rep. 2:928. 10.1038/srep00928 23226594 PMC3514644

[B32] Markram H. Muller E. Ramaswamy S. Reimann M. W. Abdellah M. Sanchez C. A.. (2015). Reconstruction and simulation of neocortical microcircuitry. Cell 163, 456–492. 10.1016/j.cell.2015.09.029 26451489

[B33] Megias M. Emri Z. Freund T. F. Gulyás A. I. (2001). Total number and distribution of inhibitory and excitatory synapses on hippocampal CA1 pyramidal cells. Neuroscience 102, 527–540. 10.1016/S0306-4522(00)00496-6 11226691

[B34] Mulders W. H. A. M. West M. J. Slomianka L. (1997). Neuron numbers in the presubiculum, parasubiculum, and entorhinal area of the rat. J. Comp. Neurol. 385, 83–94. 10.1002/(SICI)1096-9861(19970818)385:1<83::AID-CNE5>3.0.CO;2-8 9268118

[B35] Papaleonidopoulos V. Trompoukis G. Koutsoumpa A. Papatheodoropoulos C. (2017). A gradient of frequency-dependent synaptic properties along the longitudinal hippocampal axis. BMC Neurosci. 18:79. 10.1186/s12868-017-0398-4 29233091 PMC5727934

[B36] Perez-Rosello T. Baker J. L. Ferrante M. Iyengar S. Ascoli G. A. Barrionuevo G. (2011). Passive and active shaping of unitary responses from associational/commissural and perforant path synapses in hippocampal CA3 pyramidal cells. J. Comput. Neurosci. 31, 159–182. 10.1007/s10827-010-0303-y 21207127 PMC3560390

[B37] Renart A. de la Rocha J. Bartho P. Hollender L. Parga N. Reyes A.. (2010). The asynchronous state in cortical circuits. Science 327, 587–590. 10.1126/science.1179850 20110507 PMC2861483

[B38] Rosenbaum R. Smith M. A. Kohn A. Rubin J. E. Doiron B. (2016). The spatial structure of correlated neuronal variability. Nat. Neurosci. 20, 107–114. 10.1038/nn.4433 27798630 PMC5191923

[B39] Rosenbaum R. Trousdale J. Josić K. (2011). The effects of pooling on spike train correlations. Front. Neurosci. 5:58. 10.3389/fnins.2011.00058 21687787 PMC3096837

[B40] Safavi S. Dwarakanath A. Kapoor V. Werner J. Hatsopoulos N. G. Logothetis N. K.. (2018). Nonmonotonic spatial structure of interneuronal correlations in prefrontal microcircuits. Proc. Natl. Acad. Sci. U. S. A. 115, E3539–E3548. 10.1073/pnas.1802356115 29588415 PMC5899496

[B41] Scanziani M. Gahwiler B. Thompson S. (1993). Presynaptic inhibition of excitatory synaptic transmission mediated by alpha adrenergic receptors in area CA3 of the rat hippocampus *in vitro*. J. Neurosci. 13, 5393–5401. 10.1523/JNEUROSCI.13-12-05393.1993 7504723 PMC6576403

[B42] Schneider C. J. Bezaire M. Soltesz I. (2012). Toward a full-scale computational model of the rat dentate gyrus. Front. Neural Circuits 6:83. 10.3389/fncir.2012.00083 23162433 PMC3499761

[B43] Schneidman E. Berry M. J. II. Segev R. Bialek W. (2006). Weak pairwise correlations imply strongly correlated network states in a neural population. Nature 440, 1007–1012. 10.1038/nature04701 16625187 PMC1785327

[B44] Senk J. Hagen E. van Albada S. J. Diesmann M. (2018). Reconciliation of weak pairwise spike-train correlations and highly coherent local field potentials across space. arXiv arXiv:1805.10235. 10.1093/cercor/bhae405 PMC11513197 39462814

[B45] Small S. A. (2002). The longitudinal axis of the hippocampal formation: its anatomy, circuitry, and role in cognitive function. Rev. Neurosci. 13, 183–194. 10.1515/REVNEURO.2002.13.2.183 12160261

[B46] Strange B. A. Witter M. P. Lein E. S. Moser E. I. (2014). Functional organization of the hippocampal longitudinal axis. Nat. Rev. Neurosci. 15, 655–669. 10.1038/nrn3785 25234264

[B47] Swanson L. W. Wyss J. M. Cowan W. M. (1978). An autoradiographic study of the organization of intrahippocampal association pathways in the rat. J. Comp. Neurol. 181, 681–715. 10.1002/cne.901810402 690280

[B48] Tamamaki N. (1997). Organization of the entorhinal projection to the rat dentate gyrus revealed by Dil anterograde labeling. Exp. Brain Res. 116, 250–258. 10.1007/PL00005753 9348124

[B49] Tetzlaff T. Rotter S. Stark E. Abeles M. Aertsen A. Diesmann M. (2008). Dependence of neuronal correlations on filter characteristics and marginal spike train statistics. Neural Comput. 20, 2133–2184. 10.1162/neco.2008.05-07-525 18439140

[B50] Tielen A. M. Lopes da Silva F. H. Mollevanger W. J. (1981). Differential conduction velocities in perforant path fibres in guinea pig. Exp. Brain Res. 42, 231–233. 10.1007/BF00236913 7262219

[B51] Urban N. N. Henze D. A. Barrionuevo G. (2001). Revisiting the role of the hippocampal mossy fiber synapse. Hippocampus 11, 408–417. 10.1002/hipo.1055 11530845

[B52] Yassa M. A. Stark C. E. L. (2011). Pattern separation in the hippocampus. Trends Neurosci. 34, 515–525. 10.1016/j.tins.2011.06.006 21788086 PMC3183227

[B53] Yeckel M. F. Berger T. W. (1990). Feedforward excitation of the hippocampus by afferents from the entorhinal cortex: redefinition of the role of the trisynaptic pathway. Proc. Natl. Acad. Sci. 87, 5832–5836. 10.1073/pnas.87.15.5832 2377621 PMC54422

[B54] Yu G. J. Bouteiller J.-M. C. Song D. Berger T. W. (2019). Axonal anatomy optimizes spatial encoding in the rat entorhinal-dentate system: a computational study. IEEE Trans. Biomed. Eng. 66, 2728–2739. 10.1109/TBME.2019.2894410 30676938 PMC6642851

[B55] Yu G. J. Hendrickson P. J. Song D. Berger T. W. (2015). “Topography-dependent spatio-temporal correlations in the entorhinal-dentate-CA3 circuit in a large-scale computational model of the Rat Hippocampus,” in 2015 37th Annual International Conference of the IEEE Engineering in Medicine and Biology Society (EMBC). 10.1109/EMBC.2015.7319262 PMC4858183 26737162

[B56] Yu G. J. Hendrickson P. J. Song D. Berger T. W. (2018). “Spatiotemporal patterns of granule cell activity revealed by a large-scale, biologically realistic model of the hippocampal dentate gyrus,” in Springer Series in Computational Neuroscience, eds V. Cutsuridis, B. P. Graham, S. Cobb, and I. Vida (Cham: Springer International Publishing), 473–508.

[B57] Yu G. J. Song D. Berger T. W. (2014). “Implementation of the excitatory entorhinal-dentate-CA3 topography in a large-scale computational model of the rat hippocampus,” in 2014 36th Annual International Conference of the IEEE Engineering in Medicine and Biology Society (EMBC). 10.1109/EMBC.2014.6945136 PMC4494103 25571504

[B58] Zador A. Koch C. Brown T. H. (1990). Biophysical model of a Hebbian synapse. Proc. Natl. Acad. Sci. 87, 6718–6722. 10.1073/pnas.87.17.6718 2168555 PMC54608

