# Supplementary material for: Topographic Organization of Correlation Along the Longitudinal and Transverse Axes in Rat Hippocampal CA3 Due to Excitatory Afferents
Source: Front Comput Neurosci. 2020 Nov 20;14:588881. doi: 10.3389/fncom.2020.588881 (PMC7715032; doi:10.3389/fncom.2020.588881)
Supplement: Supplementary file 1 [file Table_1.DOCX]

Supplementary Material

# Supplementary Data

**Table S1.** Conductance parameters for ion channels in the bursting CA3 pyramidal cell model. Units are in S-cm^-2^.

|  | Lacunosum  Distal | Lacunosum  Proximal | Radiatum | Lucidum | Soma | Axon | Oriens  Proximal | Oriens  Distal |
| --- | --- | --- | --- | --- | --- | --- | --- | --- |
| HCN | 3.8e-5 | 4.04e-5 | 4.11e-5 | 1.28e-5 | 1e-5 | - | 7.1e-5 | 6.0e-5 |
| Na^+^ | 0.083 | 0.089 | 0.090 | 0.028 | 0.44 | 0.11 | 0.16 | 0.13 |
| Delayed-rectifier K^+^ | 0.019 | 0.020 | 0.021 | 0.0064 | 0.077 | 0.005 | 0.035 | 0.030 |
| Fast-inactivating A-type K^+^ | 0.076 | 0.081 | 0.082 | 0.026 | 0.047 | 0.02 | 0.14 | 0.12 |
| KCNQ/M-current | – | – | – | – | 0.026 | – | – | – |
| Slow-inactivating D-type K^+^ | – | – | – | – | 0.0 | – | – | – |
| L-type Ca^2+^ | – | – | – | – | 1.1e-4 | – | 7.1e-5 | 6.0e-5 |
| N-type Ca^2+^ | – | – | – | – | 1.1e-4 | – | 7.1e-5 | 6.0e-5 |
| T-type Ca^2+^ | – | – | – | – | 1.1e-4 | – | 7.1e-5 | 6.0e-5 |
| Ca^2+^-dependent K^+^ (CaGK) | – | – | – | – | 3.0e-5 | – | 3.5e-4 | 3.0e-4 |
| Ca^2+^-dependent K^+^ (BK) | – | – | – | – | 0.001 | – | 0.0071 | 0.0060 |
| Leak | 5.3e-5 | 5.6e-5 | 1.1e-4 | 3.6e-5 | 1.4e-5 | 1.4e-5 | 2.0e-04 | `1.7e-4 |

**Table S2.** Conductance parameters for ion channels in the strongly adapting CA3 pyramidal cell model. Units are in S-cm^-2^.

|  | Lacunosum  Distal | Lacunosum  Proximal | Radiatum | Lucidum | Soma | Axon | Oriens  Proximal | Oriens  Distal |
| --- | --- | --- | --- | --- | --- | --- | --- | --- |
| HCN | 3.8e-5 | 4.04e-5 | 4.11e-5 | 1.28e-5 | 1e-5 | - | 7.1e-5 | 6.0e-5 |
| Na^+^ | 0.083 | 0.089 | 0.090 | 0.028 | 0.21 | 0.11 | 0.16 | 0.13 |
| Delayed-rectifier K^+^ | 0.038 | 0.040 | 0.041 | 0.013 | 0.095 | 0.01 | 0.071 | 0.060 |
| Fast-inactivating A-type K^+^ | 0.076 | 0.081 | 0.082 | 0.026 | 0.38 | 0.02 | 0.14 | 0.12 |
| KCNQ/M-current | – | – | – | – | 0.016 | – | – | – |
| Slow- inactivating D-type K^+^ | – | – | – | – | 0.0 | – | – | – |
| L-type Ca^2+^ | – | – | – | – | 1.6e-4 | – | 7.1e-5 | 6.0e-5 |
| N-type Ca^2+^ | – | – | – | – | 1.6e-4 | – | 7.1e-5 | 6.0e-5 |
| T-type Ca^2+^ | – | – | – | – | 1.6e-4 | – | 7.1e-5 | 6.0e-5 |
| Ca^2+^-dependent K^+^ (CaGK) | – | – | – | – | 3.6e-4 | – | 1.4e-4 | 1.2e-4 |
| Ca^2+^-dependent K^+^ (BK) | – | – | – | – | 0.001 | – | 0.0071 | 0.0060 |
| Leak | 5.1e-5 | 5.4e-5 | 1.1e-4 | 3.4e-5 | 1.34e-5 | 1.34e-5 | 1.9e-04 | 1.6e-4 |

**Table S3.** Conductance parameters for ion channels in the weakly adapting CA3 pyramidal cell model. Units are in S-cm^-2^.

|  | Lacunosum  Distal | Lacunosum  Proximal | Radiatum | Lucidum | Soma | Axon | Oriens  Proximal | Oriens  Distal |
| --- | --- | --- | --- | --- | --- | --- | --- | --- |
| HCN | 3.79e-5 | 4.04e-5 | 4.11e-5 | 1.28e-5 | 1e-5 | - | 7.1e-5 | 6.0e-5 |
| Na^+^ | 0.083 | 0.089 | 0.090 | 0.028 | 0.13 | 0.11 | 0.16 | 0.13 |
| Delayed-rectifier K^+^ | 0.019 | 0.040 | 0.041 | 0.013 | 0.16 | 0.01 | 0.071 | 0.060 |
| fast-inactivating A-type K^+^ | 0.076 | 0.081 | 0.082 | 0.026 | 0.097 | 0.02 | 0.14 | 0.12 |
| KCNQ/M-current | – | – | – | – | 0.012 | – | – | – |
| Slowly inactivating D-type K^+^ | – | – | – | – | 0.0 | – | – | – |
| L-type Ca^2+^ | – | – | – | – | 3.5e-5 | – | 7.1e-5 | 6.0e-5 |
| N-type Ca^2+^ | – | – | – | – | 3.5e-5 | – | 7.1e-5 | 6.0e-5 |
| T-type Ca^2+^ | – | – | – | – | 3.5e-5 | – | 7.1e-5 | 6.0e-5 |
| Ca^2+^-dependent K^+^ (CaGK) | – | – | – | – | 2.5e-4 | – | 3.5e-4 | 3.0e-4 |
| Ca^2+^-dependent K^+^ (BK) | – | – | – | – | 0.001 | – | 0.0071 | 0.0060 |
| Leak | 5.5e-5 | 5.9e-5 | 1.2e-4 | 3.7e-5 | 1.45e-5 | 1.45e-5 | 2.0e-04 | 1.8e-4 |

**Table S4.** Passive properties and reversal potentials for all CA3 pyramidal cell models.

|  | Lacunosum  Distal | Lacunosum  Proximal | Radiatum | Lucidum | Soma | Axon | Oriens  Proximal | Oriens  Distal |
| --- | --- | --- | --- | --- | --- | --- | --- | --- |
| Length (µm) | 77.39 | 148.36 | 303.46 | 97.27 | 11.22 | 97.09 | 189.25 | 71.68 |
| Diameter (µm) | 0.89 | 1.15 | 2.95 | 6.41 | 13.21 | 1.03 | 2.55 | 1.77 |
| R_axial_ (Ω-cm) | 77.75 | 42.84 | 129.28 | 140.0 | 140.0 | 50.0 | 135.69 | 135.66 |
| c_m_ (µF-cm^-2^) | 2.73 | 2.91 | 5.92 | 1.84 | 0.72 | 0.72 | 10.17 | 8.70 |
| e_HCN_ (mV) | -30.0 | -30.0 | -30.0 | -30.0 | -30.0 | - | -30.0 | -30.0 |
| e_na_ (mV) | 55.0 | 55.0 | 55.0 | 55.0 | 55.0 | 55.0 | 55.0 | 55.0 |
| e_K_ (mV) | -90.0 | -90.0 | -90.0 | -90.0 | -90.0 | -90.0 | -90.0 | -90.0 |
| e_leak_ (mV) | -65.0 | -65.0 | -65.0 | -65.0 | -65.0 | -65.0 | -65.0 | -65.0 |

**Table S5.** AMPAR parameters for the bursting CA3 pyramidal cell model.

|  | Lacunosum  Distal  (LEC) | Lacunosum  Proximal  (MEC) | Radiatum  (Associational) | Lucidum  (Mossy Fiber) | Oriens  Proximal (Mossy Fiber) | Oriens  (Associational) |
| --- | --- | --- | --- | --- | --- | --- |
| Peak (mV) | 0.30^1^ | 0.30^1^ | 0.30^1^ | 3.22^2^ | 3.2^2^ | 0.3^1^ |
| HHW (ms) | 45.7^1^ | 45.75^1^ | 40.4^1^ | 135.9^3^ | 135.55^3^ | 39.7^1^ |
| Conductance (µS) | 3.12e-4 | 2.99e-4 | 3.60e-4 | 1.05e-3 | 1.17e-3 | 3.60e-4 |
| AMPA $\tau_{1}$ (ms) | 0.5 | 0.5 | 0.5 | 0.5 | 0.5 | 0.5 |
| AMPA $\tau_{2}$ (ms) | 12.66 | 12.66 | 9.77 | 144.03 | 144.03 | 8.77 |

^1^(Perez-Rosello et al. 2011)

^2^(Lawrence, Grinspan, and McBain 2004)

^3^(Scanziani, Gahwiler, and Thompson 1993)

**Table S6.** AMPAR parameters for the strongly adapting CA3 pyramidal cell model.

|  | Lacunosum  Distal  (LEC) | Lacunosum  Proximal  (MEC) | Radiatum  (Associational) | Lucidum  (Mossy Fiber) | Oriens  Proximal (Mossy Fiber) | Oriens  (Associational) |
| --- | --- | --- | --- | --- | --- | --- |
| Peak (mV) | 0.30^1^ | 0.30^1^ | 0.30^1^ | 3.22^2^ | 3.2^2^ | 0.3^1^ |
| HHW (ms) | 46.05^1^ | 46.1^1^ | 39.85^1^ | 134.95^3^ | 136.2^3^ | 39.75^1^ |
| Conductance (µS) | 3.18e-4 | 3.12e-4 | 3.85e-4 | 1.02e-3 | 1.15e-3 | 3.79e-4 |
| AMPA $\tau_{1}$ (ms) | 0.5 | 0.5 | 0.5 | 0.5 | 0.5 | 0.5 |
| AMPA $\tau_{2}$ (ms) | 11.88 | 11.88 | 7.61 | 128.48 | 131.59 | 8.00 |

^1^(Perez-Rosello et al. 2011)

^2^(Lawrence, Grinspan, and McBain 2004)

^3^(Scanziani, Gahwiler, and Thompson 1993)

**Table S7.** AMPAR parameters for the weakly adapting CA3 pyramidal cell model.

|  | Lacunosum  Distal  (LEC) | Lacunosum  Proximal  (MEC) | Radiatum  (Associational) | Lucidum  (Mossy Fiber) | Oriens  Proximal (Mossy Fiber) | Oriens  (Associational) |
| --- | --- | --- | --- | --- | --- | --- |
| Peak (mV) | 0.30^1^ | 0.30^1^ | 0.30^1^ | 3.22^2^ | 3.2^2^ | 0.3^1^ |
| HHW (ms) | 46.35^1^ | 46.4^1^ | 40.1^1^ | 134.4^3^ | 134.35^3^ | 40.0^1^ |
| Conductance (µS) | 3.05e-4 | 2.93e-4 | 3.30e-4 | 1.39e-3 | 1.56e-3 | 3.36e-4 |
| AMPA $\tau_{1}$ (ms) | 0.5 | 0.5 | 0.5 | 0.5 | 0.5 | 0.5 |
| AMPA $\tau_{2}$ (ms) | 16.55 | 16.55 | 11.49 | 122.27 | 122.27 | 11.88 |

^1^(Perez-Rosello et al. 2011)

^2^(Lawrence, Grinspan, and McBain 2004)

^3^(Scanziani, Gahwiler, and Thompson 1993)

**Table S8.** NMDAR parameters for all CA3 pyramidal cells. The ratio is the factor that is multiplied with the conductance value of the corresponding AMPAR to define the NMDAR conductance.

|  | Value |
| --- | --- |
| Ratio | 0.0676 |
| $\tau_{1}$ (ms) | 20.39 |
| $\tau_{2}$ (ms) | 26.68 |
| $\tau_{3}$ (ms) | 158.73 |
| $w$ | 0.963 |


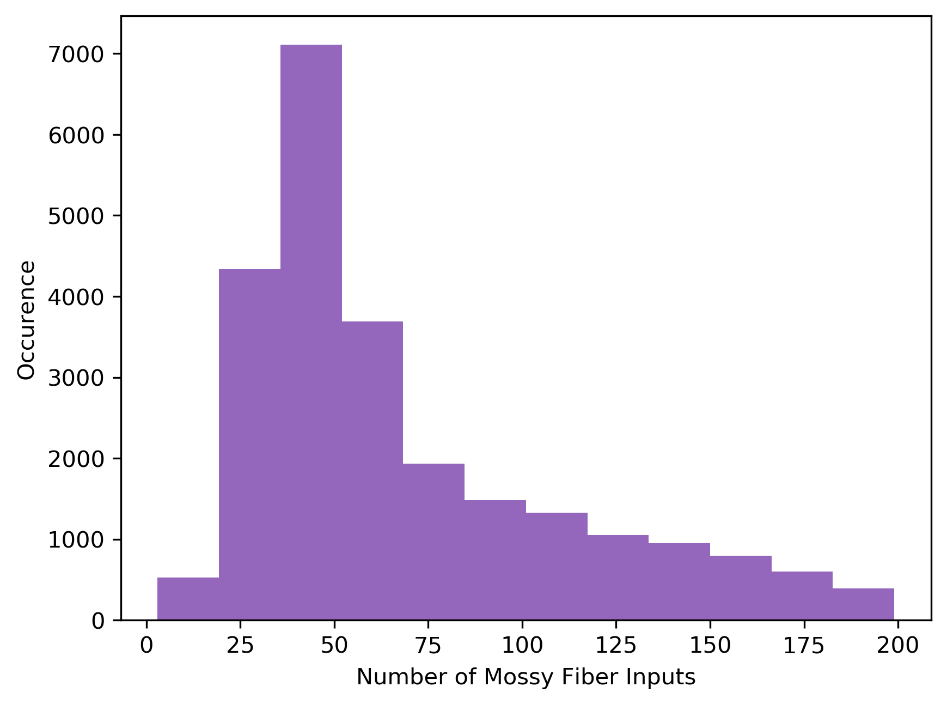


**Figure S1**. Histogram of numbers of mossy fiber inputs received by CA3 pyramidal cells. Min = 3, Max =200, Mean = 72.7, Mode = 38.8.


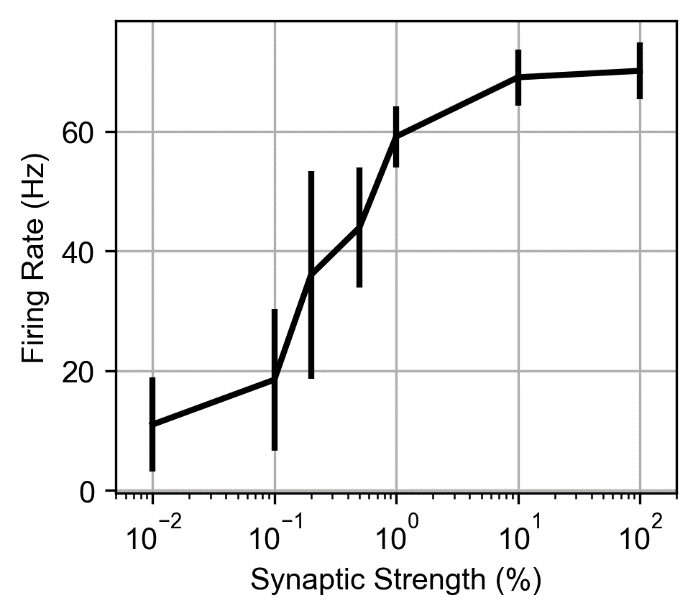


**Figure S2**. Average firing rate of CA3 pyramidal cells for varying associational synaptic strengths using the PP-wcMF-A-CA3 model. The vertical lines represent the standard deviations.

# References

Lawrence, J. Josh, Zachary M. Grinspan, and Chris J. McBain. 2004. “Quantal Transmission at Mossy Fibre Targets in the CA3 Region of the Rat Hippocampus.” *The Journal of Physiology* 554 (1): 175–93. https://doi.org/10.1113/jphysiol.2003.049551.

Perez-Rosello, Tamara, John L. Baker, Michele Ferrante, Satish Iyengar, Giorgio A. Ascoli, and Germán Barrionuevo. 2011. “Passive and Active Shaping of Unitary Responses from Associational/Commissural and Perforant Path Synapses in Hippocampal CA3 Pyramidal Cells.” *Journal of Computational Neuroscience* 31 (2): 159–82. https://doi.org/10.1007/s10827-010-0303-y.

Scanziani, M, BH Gahwiler, and SM Thompson. 1993. “Presynaptic Inhibition of Excitatory Synaptic Transmission Mediated by Alpha Adrenergic Receptors in Area CA3 of the Rat Hippocampus in Vitro.” *The Journal of Neuroscience* 13 (12): 5393–5401. https://doi.org/10.1523/JNEUROSCI.13-12-05393.1993.
